# Outreach programs to improve life circumstances and prevent further adverse developmental trajectories of at‐risk youth in OECD countries: A systematic review

**DOI:** 10.1002/cl2.1282

**Published:** 2022-10-17

**Authors:** Trine Filges, Nina T. Dalgaard, Bjørn C. A. Viinholt

**Affiliations:** ^1^ VIVE—The Danish Center for Social Science Research Copenhagen Denmark

## Abstract

**Background:**

At‐risk youth may be defined as a diverse group of young people in unstable life circumstances, who are currently experiencing or are at risk of developing one or more serious problems. At‐risk youth are often very unlikely to seek out help for themselves within the established venues, as their adverse developmental trajectories have installed a lack of trust in authorities such as child protection agencies and social workers. To help this population, a number of outreach programmes have been established seeking to help the young people on an ad hoc basis, meaning that the interventions are designed to fit the individual needs of each young person rather than as a one‐size‐fits‐all treatment model. The intervention in this review is targeted outreach work which may be (but does not have to be) multicomponent programmes in which outreach may be combined with other services.

**Objectives:**

The main objective of this review was to answer the following research questions: What are the effects of outreach programmes on problem/high‐risk behaviour of young people between 8 and 25 years of age living in OECD countries? Are they less likely to experience an adverse outcome such as school failure or drop‐out, runaway and homelessness, substance and/or alcohol abuse, unemployment, long‐term poverty, delinquency and more serious criminal behaviour?

**Search Methods:**

We identified relevant studies through electronic searches of bibliographic databases, governmental and grey literature repositories, hand search in specific targeted journals, citation tracking, and Internet search engines. The database searches were carried out in September 2020 and other resources were searched in October and November 2021. We searched to identify both published and unpublished literature, and reference lists of included studies and relevant reviews were searched.

**Selection Criteria:**

The intervention was targeted outreach work which may have been combined with other services. Young people between 8 and 25 years of age living in OECD countries, who either have experienced or is at‐risk of experiencing an adverse outcome were eligible. Our primary focus was on measures of problem/high‐risk behaviour and a secondary focus was on social and emotional outcomes. All study designs that used a well‐defined control group were eligible for inclusion. Studies that utilised qualitative approaches were not included.

**Data Collection and Analysis:**

The total number of potentially relevant studies constituted 17,659 hits. A total of 16 studies (17 different interventions) met the inclusion criteria. Only five studies could be used in the data synthesis. Eight studies could not be used in the data synthesis as they were judged to have critical risk of bias and, in accordance with the protocol, were excluded from the meta‐analysis on the basis that they would be more likely to mislead than inform. Two studies (three interventions) did not provide enough information enabling us to calculate an effect size and standard error, and one study did not provide enough information to assess risk of bias. Meta‐analysis of all outcomes were conducted on each conceptual outcome separately. All analyses were inverse variance weighted using random effects statistical models incorporating both the sampling variance and between study variance components into the study level weights. Random effects weighted mean effect sizes were calculated using 95% confidence intervals. Too few studies were included to carry out any sensitivity analyses.

**Main Results:**

Four of the five studies used for meta analysis were from the USA and one was from Canada. The timespan in which included studies were carried out was 32 years, from 1985 to 2017; on average the intervention year was 2005. The average number of participants in the analysed interventions was 116, ranging from 30 to 346 and the average number of controls was 81, ranging from 32 to 321. At most, the results from two studies could be pooled in a single meta‐analysis. It was only possible to pool the outcomes drug (other than marijuana) use, marijuana use and alcohol use each at two different time points (one and 3 months follow up). At 1 month follow up the weighted averages varied between zero and 0.05 and at 3 months follow up between −0.17 and 0.07. None of them were statistically significant. In addition, a number of other outcomes were reported in a single study only.

**Authors' Conclusions:**

Overall, there were too few studies included in any of the meta‐analyses in order for us to draw any conclusion concerning the effectiveness of outreach. The vast majority of studies were undertaken in the USA. The dominance of the USA as the main country in which outreach interventions meeting our inclusion criteria have been evaluated using rigorous methods and within our specific parameters clearly limits the generalisability of the findings. None of the studies, however, was considered to be of overall high quality in our risk of bias assessment and the process of excluding studies with critical risk of bias from the meta‐analysis applied in this review left us with only five of a total of 16 possible studies to synthesise. Further, because too few studies reported results on the same type of outcome at most two studies could be combined in a particular meta‐analysis. Given the limited number of rigorous studies available from countries other than the USA, it would be natural to consider conducting a series of randomised controlled trials evaluating the effectiveness of outreach for at‐risk youth in countries outside the USA. The trial(s) should be designed, conducted and reported according to methodological criteria for rigour in respect of internal and external validity to achieve robust results and preferably reporting a larger number of outcomes.

## 1 PLAIN LANGUAGE SUMMARY

### 1.1 Evidence on the effectiveness of outreach programmes for at‐risk youth in OECD countries is inconclusive

The evidence on outreach programmes to improve life circumstances and prevent further adverse developmental trajectories of at‐risk youth in OECD countries is inconclusive.

In this review, we aimed to find evidence of the effectiveness of outreach programmes on improving at‐risk youth's life circumstances. However, the evidence is inconclusive because of the small number of studies.

### 1.2 What is this review about?

At‐risk youth are defined as a diverse group of young people in unstable life circumstances, who are currently experiencing, or at risk of developing, one or more serious problems. At‐risk youth are often very unlikely to seek out help for themselves within the established facilities, as their adverse developmental trajectories have installed a lack of trust in authorities.

A number of outreach programmes have been established seeking to help these young people on an ad hoc basis, meaning that the interventions are designed to fit the individual needs of each young person rather than as a one‐size‐fits‐all treatment model.

**What is the aim of this review?**

This Campbell systematic review examines the effects of outreach programmes on problem/high‐risk behaviour of young people between eight and 25 year old, living in OECD countries. The review summarises evidence from five studies undertaken in the USA and Canada that involved 578 participants in total.

### 1.3 What studies are included?

Included studies had to examine the impact of targeted outreach programmes on at‐risk youth. Studies had to have a comparison group.

Sixteen studies analysing 17 different interventions were identified. Of these, only five studies could be used in the data synthesis. The studies were from the USA and Canada. There were four randomised controlled trials (RCTs) and 12 non‐randomised studies. The studies contained data for 578 participants.

### 1.4 What are the main findings of this review?

The evidence was inconclusive. At most, the results from two studies could be pooled in a single meta‐analysis. The outcomes drug (other than marijuana) use, marijuana use and alcohol use each at two different time points (one and three months follow up) were meta‐analysed. In addition, a number of other outcomes were reported in a single study only.

### 1.5 What do the findings of the review mean?

The current landscape of research on outreach programmes targeting at‐risk youth in the OECD countries shows that it has yet to be evaluated thoroughly. The evidence was inconclusive because too few studies reported results on the same type of outcome.

Furthermore, all the available evidence used in the data synthesis was from the USA and Canada, and so the findings may not be generalisable to other settings and systems outside Northern America.

None of the studies used in the meta‐analyses reported on long term impacts.

These considerations point to the need for more rigorously‐conducted studies reporting a larger number of outcomes.

### 1.6 How up‐to‐date is this review?

The review authors searched for studies published up to November 2021.

## 2 BACKGROUND

### 2.1 Description of the condition

At‐risk youth may be defined as a diverse group of young people in unstable life circumstances, who are currently experiencing or are at risk of developing one or more serious problems such as school failure or drop‐out, mental health disorders, substance and/or alcohol abuse, unemployment, long‐term poverty, delinquency and more serious criminal behaviour (Arbreton et al., 2005; Quinn, 1999). At‐risk youth typically have a multitude of social and psychological problems and typically also come from families considered at‐risk (Treskon, 2016). They may occasionally or permanently be homeless and spend time in the streets.

No readily available statistics on the numbers of at‐risk youth exist but statistics on the numbers experiencing the adverse outcomes can be found. For example, according to the National Conference of State Legislatures (NCSL) on any given night, approximately 41,000 unaccompanied youth ages 13‐25 experience homelessness in the US (NCSL, 2019). It is estimated that 4.2 million youth and young adults experience homelessness each year, and that 10% of young adults ages 18–25, and at least one in 30 adolescents ages 13–17, experience some form of homelessness over the course of a year (NCSL, 2019). A substantial part of them report having a number of other problems too; for example having substance misuse problems (29%), mental health problems (69%) or been in the juvenile justice system, in jail or detention (50%), Further, school drop‐out and no high school diploma or General Equivalency Diploma (GED) is the number one correlate for elevated risk of youth homelessness (NCSL, 2019). In Denmark the numbers are much lower. The estimated number of homeless youth, less than 25 years of age, was 1036 in 2019 (Benjaminsen, 2019) which amounts to less than 1% of those aged 13–24 years; but in line with the evidence from the US a large part of them have other problems (e.g., substance misuse and mental health problems) as well and the majority in the age group 18–24 are NEET, that is, neither employed nor in education or training (Benjaminsen et al., 2020). Numbers of homeless youth across OECD countries are hard to locate and definitions of homelessness vary across countries (OECD, 2020a) but most likely, there is as great variation as in other indicators of at‐risk youth. For example, the rates of school drop‐out, those that do not reach a basic minimum level of skills, is on average 19% across OECD countries and range from 2% in Korea to 58% in Turkey for the 25–34 years‐old (OECD, 2012). Also, the NEET rates vary a lot across OECD countries; from less than 7% of the 15–29 year old in Iceland and the Netherlands to more than 37% in South Africa with an OECD average of 13% (OECD, 2020b).

At‐risk youth are often very unlikely to seek out help for themselves within the established venues, as their adverse developmental trajectories have installed a lack of trust in authorities such as child protection agencies and social workers (Ronel, 2006). To help this population, a number of outreach programmes have been established seeking to help the young people on an ad hoc basis, meaning that the interventions are designed to fit the individual needs of each young person rather than as a one‐size‐fits‐all treatment model (Korf et al., 1999; Svensson, 2003). The programmes are often multicomponent interventions and often rely on volunteers as outreach workers, as these are proposed to offer the young people a unique possibility for forming trusting relationships due to the fact that help is offered as an act of altruism (Ronel, 2006). The programmes may offer basic necessities such as food or shelter, and they may offer counselling, mentoring and medical assistance. What define the outreach programmes is that they are targeted at helping the young people away from the streets and their current adverse developmental paths towards more stable living situations and developmental prospects.

Due to the very nature of the programmes, the effects are difficult to determine. First, randomisation is difficult when there is no system of referral, and the uniquely tailored interventions, which each young person receives raises the question if one can even describe the intervention as uniform even within the same programme. Second, the aims of the programmes are typically to change the long‐term developmental paths of the participants, but longitudinal studies are often not feasible, and the establishment of long‐term preventive effects is difficult. However, even if the obstacles are many, it is still important to explore the efficacy of outreach programmes, as the stakes are extremely high. If left alone, the target population of at‐risk youth are likely to develop serious long‐term problems, which are not just detrimental to the individual but also very costly to societies.

### 2.2 Description of the intervention

The intervention in this review is targeted outreach work which may be (but does not have to be) multicomponent programmes in which outreach may be combined with other services. There are different meanings of the concept outreach work throughout Europe and a wide variety of outreach initiatives with different arrangements where outreach may work in one or many ways (Svensson, 2003).

The term *outreach work* as we will use it in this review is commonly known throughout Scandinavia and is corresponding with detached youth work in England (similar to street work or fieldwork, Korf et al., 1999). Detached outreach work is executed outside any agency setting, is taking place in the community where groups of marginalised youth are known to meet, with the aim of engaging young people who lack any kind of belonging by directing young people to treatment or care services when necessary. It may be based on voluntary efforts, peer groups or professionals, social workers, social pedagogical workers and health workers but the common nature is to meet the young people on their own terms. Outreach work is based on voluntary participation and is an important approach for intervening with hard to reach populations, and identifying their needs in a flexible and responsive manner with no manual‐based restrictions.

However, an outreach programme may be associated with a specific service or combination of services offered by one or more organisations targeting a specific population. The services combined with the outreach component could be case management or participation in community programmes or even a continuum of comprehensive services including education, employment, and intensive supervision.

Outreach efforts with services only focusing on nutritional and medical care (e.g., testing for HIV) was excluded.

The comparison population were young people at‐risk who are not contacted by the outreach workers and are not encouraged to attend any services.

### 2.3 How the intervention might work

The primary mechanism of change in outreach work with at‐risk youth is to facilitate positive change by gradually building up a sense of trust between the young person and the outreach worker(s) (Svensson, 2003). Characteristically, the aim of the outreach youth worker is to find solutions to young people's problems in their own environment, rather than deciding while sitting behind a desk what they consider best for the person concerned. The goal is always to prevent further marginalisation and encourage social integration (Svensson, 2003).

Theoretically, outreach work may be understood through an empowerment lens. Empowerment theory is both a value orientation for working in the community and a theoretical model for understanding the processes whereby individuals gain access to resources and acquire skills and knowledge enabling them to take advantage of opportunities within the community and to exert control and influence over decisions that affect their lives (Zimmerman, 2002). As a value orientation empowerment theory proposes that many social problems exist because of unequal distribution of, and access to, resources within the community. The theory further suggests that many individuals are best served by mutual help, helping others or working for their rights rather than having their needs fulfilled by a benevolent professional (Perkins & Zimmerman, 1995; Zimmerman, 2002). What this means is that outreach work is aimed at enabling the at‐risk young person to function more autonomously and adaptively within their community rather than just providing a quick fix for their current problems. Empowerment theory proposes that by identifying strengths rather than pointing out and cataloguing risk factors, at‐risk youth may become motivated to actively engage in their own positive change. Outreach work may thus also be understood as aimed at promoting resiliency by enabling the young person to make better use of their personal and social resources. Theoretically a number of protective factors may serve to buffer the adversity a young person might be exposed to. Protective factors at the personal level may include being physically healthy, having a good self‐esteem and adaptive coping skills. At the family level protective factors may include having a supportive network of family or friends and at the societal level protective factors may include living in a community with access to support. Thus, outreach work may be seen as drawing on resiliency theory when working to assist the young person in identifying protective factors (Zimmerman et al., 2013). As proposed by Rappaport (1985) social change based on empowerment is proposed to be brought on by a change of both language and conceptions. Instead of perceiving the outreach workers and at‐risk young people as ‘professionals’ and ‘clients’, empowerment thinking proposes a bidirectional relationship between helpers and participants. In outreach work this means that the outreach workers aim to meet the at‐risk youth with a non‐judgemental approach characterised by genuine empathy rather than prejudice and victim blaming (Svensson, 2003; Zimmerman, 2002). In addition to meeting the youth with empathy outreach workers strive to become ‘culturally competent’ which may be defined as the willingness to understand young people from different cultural and social backgrounds and the ability to put oneself in their situation. It also includes the ability and readiness to sympathise with young people subjected to prejudice, social exclusion and stigmatisation, and to approach each young person with respect, open‐mindedness and commitment (Svensson, 2003).

As stated in the introduction at‐risk youth often come from socio‐economically less advantaged and dysfunctional families (Treskon, 2016). At risk youth have often experienced at number of adverse events such as poverty, emotional or physical abuse and neglect, out‐of‐home placement, living with mentally ill or substance abusing parents and unstable housing situations leading to a lack of continuity in their education. Thus, at‐risk youth often lack stable attachment figures and suitable adult role models, which leads to a lack of adaptive life skills and compromises their ability to seek appropriate help within established venues. Early adverse experiences may also lead to a deeply installed mistrust of authorities and thus at‐risk youth are often unlikely to seek out help for themselves. In line with empowerment thinking, outreach programmes seek to meet the young person at their own terms offering them the specific help they need here and now and thus slowly building up a trusting relationship which may be used for future motivational work (Svensson, 2003). Outreach workers aim at offering the young person a positive adult role model and thus provide the young person with the kind of socio emotional support which they often lack. Sometimes outreach workers may teach the young person basic life skills, such as personal hygiene, offer assistance with homework or writing job applications, paying bills, getting help for substance or alcohol abuse problems and being on time for work or school, or they may accompany the young person to meetings with authority figures, which are fear‐inducing in the young person due to their negative past experiences. Furthermore, outreach work may include tutoring programmes, or offer assistance with baby‐sitting and housing for socially disadvantaged teenage mothers. What characterises all efforts is that they seek to support and instal a sense of empowerment within the young person which may enable them to master similar challenges in the future in a more adaptive way and to motivate the young person to behaviour changes which may facilitate further social re‐integration (Perkins & Zimmerman, 1995; Svensson, 2003; Zimmerman, 2002).

In sum, empowerment theory provides a framework for understanding the mechanisms of change within youth outreach work. The goal of outreach work with at‐risk youth is to facilitate positive long‐term social change by motivating the young person to become actively engaged. Based on Svensson (2003) the theoretical approach to youth outreach work is based on the following principles:

– Distribution of services where youth, subcultural groups, young people at risk and young drug users are present in their own environment.
– To design services based on the needs young people demonstrate and encourage their voluntary participation.
– The outreach work is based on voluntary relations between the youth and the outreach worker. The relation is based on confidence, distinctness and continuity.
– The outreach work is executed on the young people's own terms.
– Respect for the youth's own values, their needs, their civil and human rights, their choice and their responsibility for their own lives. Meet people with non‐judgemental attitude, integrity, frankness and honesty.

### 2.4 Why it is important to do this review

We have located one systematic review on outreach programmes for youth; however, it only included programmes for street‐involved youth, a term used by the authors instead of homeless youth (Connolly & Joly, 2012). The participant population was young people aged 12–25, who did not have a permanent place of residence. Furthermore, it only included articles published in peer‐review journals between 1990 and 2010 and had no restrictions on how the studies measured an impact (i.e., studies without comparison groups were included). The only impact result reported is on later participation rates in the offered service.

Further, we have located five systematic reviews on street‐connected and/or homeless youth.

The systematic review by Coren et al. (2016); focused on street‐connected children and young people (i.e., living on, or closely connected to, the street), from birth to 24 years, and included studies of harm reduction or reintegration interventions that used a comparison group study design. The searches were performed up to April 2015. The primary outcomes of the review were inclusion and reintegration. The secondary outcomes were measures of health, well‐being and educational and occupational achievement. Thirteen studies were included and most of them compared therapy‐based services versus usual shelter and drop‐in services, or versus other therapeutic/health interventions.

Another systematic review on homeless youth (between the ages of 12–24 years) focused solely on HIV/acquired immunodeficiency syndrome (AIDS) prevention programmes (Naranbhai et al., 2011). The searches were performed up to December 2010 and only randomised controlled trials were included.

In the systematic review by Altena et al. (2010), studies published up to 2008 were included if they empirically examined the effectiveness of an intervention for homeless youth. Randomised as well as non‐randomised studies and studies without a control group, that is, before‐after studies were included. No meta‐analysis was performed, only a narrative analysis describing each study and results.

The systematic review by Slesnick et al. (2009), included runaway, shelter, street or drop‐in centre recruited youth between the ages of 12–24. In addition to intervention studies, the review also included studies assessing youth outcomes after shelter or drop‐in utilisation (i.e., service evaluations) and qualitative studies. No meta‐analysis was performed, only a narrative analysis describing each study and results. When the searches were performed is not reported.

In Xiang (2013), studies that examined the effectiveness of interventions to improve substance abuse problems amongst homeless youth between the ages of 12 and 24 were included. Searches were performed up to April 2012. Only studies that reported data on substance use outcomes were included. Randomised as well as non‐randomised studies and studies without a control group, that is, before‐after studies were included. No meta‐analysis was performed, only a narrative analysis describing each study and results.

Three systematic reviews were found, focusing explicitly on mentoring interventions for youth.

Tolan et al. (2008) performed a systematic review on mentoring intervention with the aim of affecting juvenile delinquency and associated problems for youth, defined as persons under age 18. The review was limited to studies conducted within the United States or another predominately English‐speaking country reported between 1970 and 2005. Eligible outcomes were measures of juvenile delinquency, aggression or high levels of externalising problems, drug abuse and academic achievement/school failure.

DuBois et al. (2002) searched for studies from 1970 through 1998 reporting on the effectiveness of one‐on‐one mentoring programmes for youth. The eligible age of youth is not reported but the average age of the youth participants in the study population had to be less than 19. The review included before‐after studies, and excluded studies were the adult mentors were mental health professionals (e.g., social workers). Studies of peer tutoring or mentoring programmes were also excluded. It is unclear what the eligible outcomes were, all outcomes was analysed in one meta‐analysis; however a moderator analysis distinguishes between the outcome types: emotional/psychological, problem/high‐risk behaviour, social competence, academic/educational and career/employment.

DuBois et al. (2011) is a follow‐up to DuBois et al. (2002) with some modifications. Before‐after studies were no longer eligible, participants was required to be less than 18 years, studies of peer tutoring or mentoring programmes were now eligible and mentoring was not required to be one‐on‐one. The review included studies published between 1999 and 2010. Eligible outcomes were attitudinal/motivational, social/relational, psychological/emotional, conduct problems, academic/school, physical health, and career/employment.

Besides being up‐to‐date, a major difference between these nine systematic reviews and the review we have performed is, that we focused on programmes with a targeted outreach component for youth aged 8–25. Participants need not be homeless (but were eligible if they were), and we only included studies with a control group. All relevant outcome areas were analysed separately in meta‐analyses taking into consideration the dependencies between effect sizes.

#### 2.4.1 Policy relevance

Public as well as private after‐school programmes and youth clubs that provide healthy alternatives for youth have been shown to serve as important resources for reducing school failure and youth crime (Parker, 2011). However, it is questionable whether the youth who would benefit most are those who are attracted to and attend such programmes (Arbreton & McClanahan, 2002). Outreach work represents an important preventive working approach with the aim of attracting and serving the youth who are very unlikely to participate on their own and who probably need help the most.

Outreach programmes targeting at‐risk youth are designed to reach the youth who need help to prevent high‐school dropout, crime, drug abuse, and other forms of delinquency. Besides the non‐monetary costs in terms of pain, suffering, and lost quality of life the youth incur themselves, there are potentially large financial costs to society that can be saved. A 1998 study estimated the total costs to society of allowing one youth to leave high school for a life of crime and drug abuse to be somewhere between $1.7 and $2.3 million (Cohen, 1998). There are thus more than one good reason to put more weight on prevention efforts.

## 3 OBJECTIVES

The main objective of this review was to answer the following research questions: What are the effects of outreach programmes on problem/high‐risk behaviour of young people between 8 and 25 years of age living in OECD countries? Are they less likely to experience an adverse outcome such as school failure or drop‐out, runaway and homelessness, substance and/or alcohol abuse, unemployment, long‐term poverty, delinquency and more serious criminal behaviour?

## 4 METHODS

### 4.1 Criteria for considering studies for this review

#### 4.1.1 Types of studies

The proposed project followed standard procedures for conducting systematic reviews using meta‐analysis techniques. The systematic review protocol (Filges et al., 2020) was published in December 2020. The protocol is available at: https://onlinelibrary.wiley.com/doi/10.1002/cl2.1121.

To summarise what is known about the possible causal effects of outreach, we included all study designs that use a well‐defined control group. Non‐randomised studies, where outreach has occurred in the course of usual decisions outside the researcher's control, must demonstrate pre‐treatment group equivalence via matching, statistical controls, or evidence of equivalence on key risk variables and participant characteristics. These factors were outlined in the protocol, and the methodological appropriateness of the included studies assessed according to a risk of bias model.

The study designs eligible for inclusion in the review were:

1. Controlled trials (where all parts of the study are prospective, such as identification of participants, assessment of baseline, and allocation to intervention, and which may be randomised or non‐randomised), assessment of outcomes and generation of hypotheses (Higgins & Green, 2011).
2. Non‐randomised studies (outreach has occurred in the course of usual decisions, the allocation to outreach, and no outreach is not controlled by the researcher, and there is a comparison of two or more groups of participants, that is, at least a treated group and a control group).

Non‐randomised studies using an instrumental variable approach were not eligible—see Supporting Information: Appendix 1 (*Justification of exclusion of studies using an instrumental variable (IV) approach*) for our rationale for excluding studies of these designs.

#### 4.1.2 Types of participants

Young people between 8 and 25 years of age living in OECD countries, who either have experienced or is at‐risk of experiencing an adverse outcome such as school failure or drop‐out, runaway and homelessness, substance and/or alcohol abuse, unemployment, long‐term poverty, delinquency/criminal behaviour were eligible.

At‐risk may be based on such indicators as the young person's level of association with negative peers (e.g., negative attitudes towards school and poor educational outlook, gang members, etc.), hanging out on the streets or in gang neighbourhoods, poor academic history, coming from a highly distressed or crisis ridden, low income family in a racially/ethnically segregated neighbourhood, and prior involvement in illegal and delinquent activities.

Studies where the majority of participants are between 8 and 25 years of age were not eligible.

#### 4.1.3 Types of interventions

The intervention in this review are targeted outreach work which may be combined with other services. There are different meanings of the concept outreach work throughout Europe (Svensson, 2003). The term *outreach work* as we will use it in this review is commonly known throughout Scandinavia and is corresponding with detached youth work in England (similar to street work or fieldwork, Korf et al., 1999). Detached outreach work is executed outside any agency setting, is taking place in the community where groups of marginalised youth are known to meet, with the aim of engaging young people who lack any kind of belonging, and directing young people to treatment or care services when necessary. An outreach programme may be associated with a specific service or combination of services offered by one or more organisations targeting a specific population. The services combined with the outreach component could be case management or participation in community programmes or even a continuum of comprehensive services including education, employment, and intensive supervision.

Outreach efforts with services only focusing on nutritional and medical care (e.g., testing for HIV) were excluded.

The comparison population were young people at‐risk who are not contacted and encouraged by the outreach workers to attend any services.

#### 4.1.4 Types of outcome measures

The primary outcome was problem/high‐risk behaviour, as the overall review question is to evaluate current evidence on outreach programmes' effects on problem/high‐risk behaviour for young people who have experienced or are at risk of experiencing an adverse outcome. We sought evidence on how to best reduce or eliminate problem/high‐risk behaviour, as problem/high‐risk behaviour is understood as the young people's primary problem.

All measures were included, that is, we did not require that measures have been standardised on a different population.

### 4.2 Primary outcomes

The primary focus was on measures of problem/high‐risk behaviour, such as delinquency/criminal behaviour, drug and alcohol use, high levels of externalising problems, school failure, sexual risk taking, gang involvement/membership, poverty, unemployment, runaway and homelessness.

### 4.3 Secondary outcomes

A secondary focus was on measures of social and emotional outcomes, such as internalising symptoms (anxiety, depression), self‐identity, interpersonal relations and social awareness.

#### 4.3.1 Adverse outcomes

Any adverse effects of interventions were included as an outcome including a worsening of outcome on any of the included measures.

##### Duration of follow‐up

We planned to include outcomes measured during and after intervention as well as follow‐up at any given point in time.

##### Types of settings

Detached outreach work is executed outside any agency setting, is carried out in the community where groups of marginalised youth are known to meet, with the aim of engaging young people who lack any kind of belonging, and attracting young people to treatment or care services when necessary.

Distribution of outreach services thus takes place where youth, subcultural groups, young people at risk and young drug users are present in their own environment.

Furthermore, outreach services delivered in any format meaning were eligible, that is, services that are delivered at an individual level (that includes conversation, adult contacts, following up and being available), at a group level (the outreach worker relates to different youth groups and gangs, and initiates in‐group activities) and finally local community work (such as finding places for the young people to spend their spare‐time, contact and collaboration with other youth workers and between voluntary and public organisations when that is suitable).

### 4.4 Search methods for identification of studies

We implemented a wide range of search methods and strategies to maximise coverage of relevant references, while simultaneously attempting to reduce different types of bias related to publication and dissemination systems. The different strategies and methods will be presented below.

#### 4.4.1 Electronic searches

##### Selection of bibliographical databases

We selected bibliographical databases that cover journals from different academic disciplines relating to the topic of the review. We also selected databases with a general academic scope, to ensure coverage beyond the expected academic fields. We selected the follow databases:

- ERIC (EBSCO)
- Academic Search Premier (EBSCO)
- EconLit (EBSCO)
- PsycINFO (EBSCO)
- SocIndex (EBSCO)
- International Bibliography of the Social Sciences (ProQuest)
- Sociological Abstracts (ProQuest)
- Science Citation Index Expanded (Web Of Science)
- Social Sciences Citation Index (Web Of Science)

The database searches were performed in September 2020.

##### Example of a search string

The search strings were modified according to the search interface, syntax and subject terms for each of the above standing databases. All database searches are documented in Supporting Information: Appendix 2.

##### Description and rationale for search terms and facets, and sensitivity of the search string

The search string was designed to balance sensitivity and precision. The search string contains two aspects related to the inclusion criteria of the review. To keep the search string sufficiently sensitive, we searched each aspect in either title, abstract or subject terms.

- Search 1–4 covers the intervention
- Search 5–8 covers the population characteristics
- Search 9 combines the two search facets

A full report on the search strings and results for each database search can be found in Supporting Information: Appendix 2.

*Limitations of the search string*

The supplemental and grey literature sources included American, Swedish, Danish, and Norwegian sources, but did not include specific other regional sources (Canadian, Australian, British) which may be a limitation of the review. We did not implement any language or year restrictions to the searches on bibliographical databases.

#### 4.4.2 Searching other resources

The searches on other resources and for unpublished literature was done between the 13/10/2021 and 30/11/2021. We searched a range of web‐based resources to identify references that where either unpublished, not in English, or both. Terms used to search other resources were based on the general search strategy. Combinations of terms such as outreach with terms for the population (i.e., youth or at‐risk) were utilised. All of these searches can be seen in Supporting Information: Appendix 2.

Due to the language restrictions of the review team, we selected Danish, Swedish and Norwegian as ‘other languages’ to search in, to identify relevant unpublished literature.

Some resources listed contains multiple types of unpublished literature, as well as published references. The resources we searched are listed under the category of literature that is most prevalent in the resource.

*Searches for working papers, reports and conference proceedings*

- American Institutes for Research (AIR)—https://www.air.org/
- Manpower Demonstration Research Corporation (MDRC)—https://www.mdrc.org/
- Urban Institute—https://www.urban.org/
- Public/Private Ventures (P/PV) publications—https://ppv.issuelab.org

*Searches for dissertation and theses*

- EBSCO Open Dissertations (EBSCO)
- ProQuest Dissertations and Theses (Proquest)

*Searches for non‐US working papers, conference proceedings, dissertations and theses*

- SwePub—Academic publications at Swedish universities: http://swepub.kb.se
- NORA—Norwegian Open Research Archives: http://nora.openaccess.no/
- Skolporten—Swedish Dissertations: https://www.skolporten.se/forskning/
- DIVA–Digital Scientific Archives: http://www.diva-portal.org/smash/
- Copenhagen Business School research portal: https://research.cbs.dk/
- CRISTIN (Current Research Information System In Norway)
- Dansk Institut for Internationale studier—DIIS' publikationer: https://www.diis.dk/publikationer
- Københavns Universitet forskning: http://forskning.ku.dk/find-en-forsker/
- Roskilde Universitets forskningsportal: https://forskning.ruc.dk/
- Syddansk Universitets forskningsportal: https://portal.findresearcher.sdu.dk/
- UC Viden ‐ Professionshøjskolernes Videndatabase: https://www.ucviden.dk/
- Aalborg Universitets forskningsportal: https://vbn.aau.dk/
- Aarhus Universitets forskningspublikationer: https://pure.au.dk/portal/

*Searches for working papers, conference proceedings, dissertations and theses in English and other languages*

- *CORE—research outputs from international repositories*: https://core.ac.uk/

*Internet searches for reports*

- Google—https://www.google.dk/
- Google Scholar—https://scholar.google.com/

##### Hand searches

We implemented hand searches in key journals to identify references that were poorly indexed in the bibliographical databases, as well as covering references that was published in a journal, but not yet indexed in the bibliographical databases during the search process.

Our selection of journals to hand search was based on the frequency of the journals in our pilot‐searches for designing the search‐strings in the protocol phase. Journals with the highest frequency of references in the pilot searches were selected for hand search. We searched the following journals in the time period between 2018 and September 2021:

- Children and Youth Services
- The Future of Children
- Research on Social Work Practice
- Journal of Prevention and Intervention in the Community

*Citation‐tracking and snowballing methods*

To identify both published studies and grey literature we utilised citation‐tracking/snowballing strategies. Our primary strategy was to citation‐track related systematic‐reviews and meta‐analyses. The review team also checked reference lists of included primary studies for new leads.

*Contact to experts*

By e‐mail during September 2021, we contacted international experts to identify unpublished and ongoing studies.

### 4.5 Data collection and analysis

#### 4.5.1 Criteria for determination of independent findings

To account for possible statistical dependencies, we examined a number of issues: we assessed whether individuals had undergone multiple interventions, whether there were multiple treatment groups and whether several studies were based on the same data source.

*Multiple interventions groups and multiple interventions per individuals*

There were no studies with multiple intervention groups or multiple interventions per individual.

*Multiple studies using the same sample of data*

There were no studies using the same sample of data.

*Multiple time points*

When the results were measured at multiple time points, each outcome at each time point were analysed in a separate meta‐analysis with other comparable studies taking measurements at a similar time point. The measures were all taken at different time points, one study reported at post‐intervention, one during intervention and two studies at one month and three months follow up.

#### 4.5.2 Selection of studies

**Figure 1:**
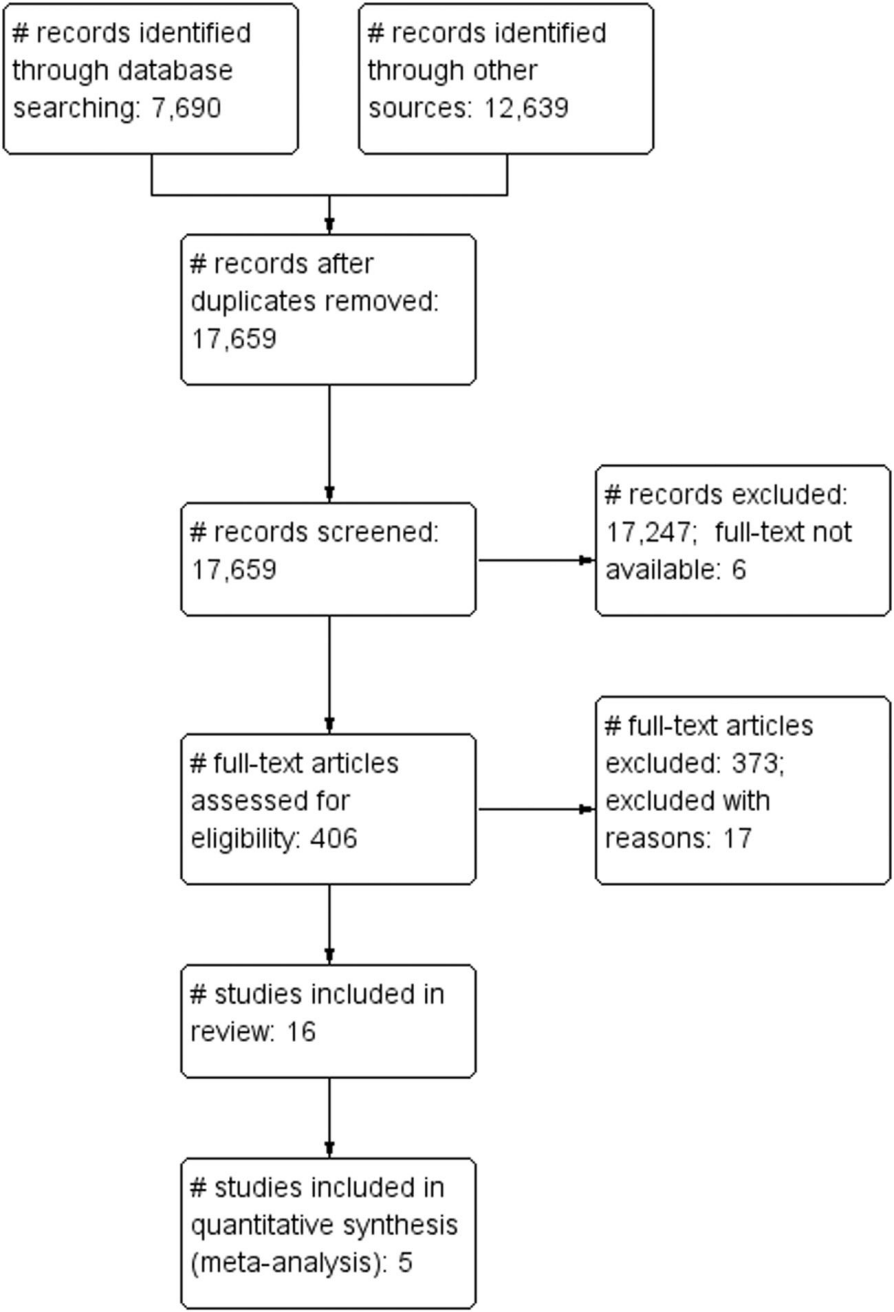
Flow diagram

Under the supervision of the review authors, two review team assistants first independently screened titles and abstracts to exclude studies that were clearly irrelevant. Studies considered eligible by at least one assistant or studies with insufficient information in the title and abstract to judge eligibility, were retrieved in full text. The full texts were then screened independently by two review team assistants under the supervision of the review authors. Any disagreement of eligibility was resolved by the review authors. Exclusion reasons for studies that otherwise might be expected to be eligible were documented and presented in section Excluded studies. The study inclusion criteria were piloted by the review authors (see Supporting Information: Appendix 3). The overall search and screening process is illustrated in Figure 1. None of the review authors were blind to the authors, institutions, or the journals responsible for the publication of the articles.

#### 4.5.3 Data extraction and management

Independent screening and deduplication of identified records was carried out in EPPI‐Reviewer 4 version 4.12.0.0.

Two review authors independently coded and extracted data from included studies.

A coding sheet was piloted on several studies and revised as necessary (see Supporting Information: Appendix 4). Disagreements were minor and were resolved by discussion. Data and information was extracted on: available characteristics of participants, intervention characteristics and control conditions, research design, sample size, risk of bias and potential confounding factors, outcomes, and results. Extracted data was stored electronically. Analysis was conducted in RevMan.

Extracted numerical and descriptive data, and the risk of bias assessments described in the next section, can be found in the supplementary documents.

#### 4.5.4 Assessment of risk of bias in included studies

We assessed the risk of bias in randomised studies using Cochrane's revised risk of bias tool, ROB 2 (Higgins et al., 2019).

The tool is structured into five domains, each with a set of signalling questions to be answered for a specific outcome. The five domains cover all types of bias that can affect results of randomised trials.

The five domains for individually randomised trials are:

(1) bias arising from the randomisation process;
(2) bias due to deviations from intended interventions (separate signalling questions for effect of assignment and adhering to intervention);
(3) bias due to missing outcome data;
(4) bias in measurement of the outcome;
(5) bias in selection of the reported result.

For cluster‐randomised trials, an additional domain was included ((1b) Bias arising from identification or recruitment of individual participants within clusters). We used the latest template for completion (currently it is the version of 15 March 2019 for individually randomised parallel‐group trials and 20 October 2016 for cluster randomised parallel‐group trials). In the cluster randomised template however, only the risk of bias due to deviation from the intended intervention (effect of assignment to intervention; intention to treat ITT) is present and the signalling question concerning the appropriateness of the analysis used to estimate the effect is missing. Therefore, for cluster randomised trials we only used the signalling questions concerning the bias arising from identification or recruitment of individual participants within clusters from the template for cluster randomised parallel‐group trials; otherwise we used the template and signalling questions for individually randomised parallel‐group trials.

We assessed the risk of bias in non‐randomised studies, using the model ROBINS–I, developed by members of the Cochrane Bias Methods Group and the Cochrane Non‐Randomised Studies Methods Group (Sterne et al., 2016a). We used the latest template for completion (currently it is the version of 19 September 2016).

The ROBINS‐I tool is based on the Cochrane RoB tool for randomised trials, which was launched in 2008 and modified in 2011 (Higgins et al., 2011).

The ROBINS‐I tool covers seven domains (each with a set of signalling questions to be answered for a specific outcome) through which bias might be introduced into non‐randomised studies:

(1) bias due to confounding
(2) bias in selection of participants
(3) bias in classification of interventions
(4) bias due to deviations from intended interventions;
(5) bias due to missing outcome data;
(6) bias in measurement of the outcome;
(7) bias in selection of the reported result.

The first two domains address issues before the start of the interventions and the third domain addresses classification of the interventions themselves. The last four domains address issues after the start of interventions and there is substantial overlap for these four domains between bias in randomised studies and bias in non‐randomised studies trials (although signalling questions are somewhat different in several places, see Sterne et al., 2016 and Higgins et al., 2019).

Randomised study outcomes are rated on a ‘Low/Some concerns/High’ scale on each domain; whereas non‐randomised study outcomes are rated on a ‘Low/Moderate/Serious/Critical/No Information’ scale on each domain. The level ‘Critical’ means: the study (outcome) is too problematic in this domain to provide any useful evidence on the effects of intervention, and it is excluded from the data synthesis. The same critical level of risk of bias (excluding the result from the data synthesis) is not directly present in the RoB 2 tool, according to the guidance to the tool (Higgins et al., 2019).

In the case of a RCT, where there is evidence that the randomisation has gone wrong or is no longer valid, we planned to assess the risk of bias of the outcome measures using ROBINS‐I instead of ROB 2. Examples of reasons for assessing RCTs using the ROBINS‐I tool may include studies showing large and systematic differences between treatment conditions while not explaining the randomisation procedure adequately suggesting that there was a problem with the randomisation process; studies with large‐scale differential attrition between conditions in the sample used to estimate the effects; or studies selectively reporting results for some part of the sample or for only some measured outcomes. In such cases, differences between the treatment and control conditions are likely systematically related to other factors than the intervention and the random assignment is, on its own, unlikely to produce unbiased estimates of the intervention effects. Therefore, as ROBINS‐I allow for an assessment of for example confounding, we believe it is more appropriate to assess effect sizes from studies with a compromised randomisation using ROBINS‐I than ROB 2. We reported this decision as part of the risk of bias assessment of the outcome measure in question (one study and all outcomes measured in this study was moved from ROB 2 to ROBINS‐I). As other effect sizes assessed with ROBINS‐I, the effect sizes could have received a ‘Critical’ rating and thus be excluded from the data synthesis.

We stopped the assessment of a non‐randomised study outcome as soon as one domain in the ROBINS‐I was judged as ‘Critical’.

‘Serious’ risk of bias in multiple domains in the ROBINS‐I assessment tool may lead to a decision of an overall judgement of ‘Critical’ risk of bias for that outcome, and it will be excluded from the data synthesis.

##### Confounding

An important part of the risk of bias assessment of non‐randomised studies is consideration of how the studies deal with confounding factors. Systematic baseline differences between groups can compromise comparability between groups. Baseline differences can be observable (e.g., age and gender) and unobservable (to the researcher; e.g., motivation and ‘ability’). There is no single non‐randomised study design that always solves the selection problem. Different designs represent different approaches to dealing with selection problems under different assumptions, and consequently require different types of data. There can be particularly great variations in how different designs deal with selection on unobservables. The ‘adequate’ method depends on the model generating participation, that is, assumptions about the nature of the process by which participants are selected into a programme.

A major difficulty in estimating causal effects of outreach work is the potential endogeneity of the young individual's life circumstance that leads to the decision of the outreach worker to reach out to that particular young person and if not accounted for it will yield biased estimates.

As there is no universal correct way to construct counterfactuals for non‐randomised designs, we looked for evidence that identification was achieved, and that the authors of the primary studies justified their choice of method in a convincing manner by discussing the assumption(s) leading to identification (the assumption(s) that make it possible to identify the counterfactual). Preferably the authors should make an effort to justify their choice of method and convince the reader that the only difference between a treated individual and a nontreated individual is the treatment. The judgement is reflected in the assessment of the confounder unobservables in the list of confounders considered important at the outset (see Supporting Information: Appendix 1, *User guide for unobservables*).

In addition to unobservables, we had identified the following observable confounding factors to be most relevant: age, gender and risk indicators as described in section *Type of participants*. In each study, we assessed whether these factors had been considered, and in addition we assessed other factors likely to be a source of confounding within the individual included studies.

##### Importance of prespecified confounding factors

The motivation for focusing on age, gender and risk indicators is given below.

The prevalence of different types of behavioural and psychological problems, coping skills, cognitive and emotional ability vary throughout a child's development through puberty and into adulthood (Cole et al., 2005), and therefore we consider age to be a potential confounding factor. Furthermore, there are substantial gender differences in behaviour problems, coping and risk of different types of adverse outcomes which is why we also include gender as a potential confounding factor (Card et al., 2008; Hampel & Petermann, 2005; Hart et al., 2007).

Pre‐treatment group equivalence of risk indicators is indisputable an important confounder as young people in stable life circumstances, typically are not at risk of developing the range of problems we will consider in this review. Therefore, the accuracy of the estimated effects of outreach programmes will depend crucially on how well the risk indicators are controlled for.

##### Effect of primary interest and important co‐interventions

We were mainly interested in the effect of starting and adhering to the intended intervention, that is, the treatment on the treated (TOT) effect. The risk of bias assessments was therefore performed in relation to this specific effect.

As the intervention is outreach to young people who are very unlikely to seek out help for themselves, we could not think of any important differences in additional interventions (‘co‐interventions’) between intervention groups that could bias the estimated effect.

##### Assessment

At least two review authors independently assessed the risk of bias for each relevant outcome from the included studies. We discussed all initial disagreements and were able to reach a consensus in all cases. We report the risk of bias assessment in risk of bias tables for each included study outcome in a supplementary document.

#### 4.5.5 Measures of treatment effect

##### Continuous outcomes

All but two outcomes (housing and NEET status) were continuous measures. We calculated effects sizes with 95% confidence intervals, where means and standard deviations were available, or alternatively from mean differences and standard deviations of the mean (whichever were available), using the methods suggested by Lipsey and Wilson (2001). If not enough information was available, we requested this information from the principal investigators. Hedges' *g* was used for estimating standardised mean differences (SMD).

##### Dichotomous outcomes

For the three dichotomous outcomes (housing, NEET status and gang membership), we used odds ratios with 95% confidence intervals.

#### 4.5.6 Unit of analysis issues

There were no studies where the unit of allocation differed from the unit of analysis.

#### 4.5.7 Dealing with missing data

Missing data and attrition rates was assessed in the included studies; see section *Assessment of risk of bias in included studies*. Where studies had missing summary data, such as missing standard deviations, we requested this information from the principal investigators. We contacted Professor Kidd who kindly forwarded our request to researcher Scott Leon who provided the necessary information. We also contacted Professor Amy Arbreton and Wendy McClanahan concerning two studies published by the now closed Public/Private Ventures. They kindly replied to our request but unfortunately the information we were seeking no longer exist.

#### 4.5.8 Assessment of heterogeneity

Heterogeneity among primary outcome studies was assessed with *χ* ^2^ (*Q*) test, and the *I* ^2^, and *τ* ^2^ statistics (Higgins et al., 2003). Any interpretation of the *χ* ^2^ test was made cautiously on account of its low statistical power.

#### 4.5.9 Assessment of reporting biases

Reporting bias refers to both publication bias and selective reporting of outcome data and results. Here, we state how we planned to assess publication bias.

We planned to use funnel plots for information about possible publication bias however we did not find sufficient studies (Higgins & Green, 2011).

#### 4.5.10 Data synthesis

Meta‐analysis of outcomes were conducted on each metric (as outlined in section ‘Types of outcomes measures’) separately.

Studies that were coded Critical risk of bias were not included in the data synthesis.

All analyses were inverse variance weighted using random effects statistical models that incorporate both the sampling variance and between study variance components into the study level weights. Random effects weighted mean effect sizes were calculated using 95% confidence intervals.

We provided a graphical display (forest plot) of effect sizes.

#### 4.5.11 Subgroup analysis and investigation of heterogeneity

There were not enough studies to perform moderator analyses.

#### 4.5.12 Sensitivity analysis

There were not enough studies to perform sensitivity analyses.

##### Treatment of qualitative research

We did not plan to include qualitative research.

## 5 RESULTS

### 5.1 Description of studies

#### 5.1.1 Results of the search

We summarised the search results in a flow chart in Figure 1. The total number of potential relevant studies was 17,659 after excluding duplicates (database: 5020, grey, hand search, snowballing and other resources: 12,639). We screened all studies based on title and abstract; 17,247 were excluded for not fulfilling the screening criteria, six studies were unobtainable despite efforts to locate them through libraries and searches on the Internet (they are listed in Table 1) and 406 studies were ordered, retrieved, and screened in full text. Of these, 390 did not fulfil the screening criteria and were excluded. We included a total of 16 studies in the review. The references are listed in section *References to included studies*.

**Table 1:** Studies not available

Author (year)	Title	Available journal/report information
Cohen, M. I., Williams, K., Bekelman, A. M., and Crosse, S. (1994)	National Evaluation of the Youth Gang Drug Prevention Program, Volume I: Final Report	Development Services Group; Inc.
Goldman, I. J., Kohn, M., Epstein, J., Geiler, I. and McDonald, R. G. (1972)	Youth & Work Training Programs ‐ An Evaluative Study	Report, New York State
Hermann Dieter (2009)	Prevention of Crime with mobile Youth Work	KRIMINALISTIK, pages 344–348
Insight Associates (1977)	Boys' Clubs of America Alcohol Abuse Prevention Project, 1976‐77. Final Evaluation.	Report, Great Neck; NY
Insight Associates (1978)	Boys' Clubs of America Alcohol Abuse Prevention Project, 1977‐78. Final Evaluation.	Report, Great Neck; NY
Kerr, N., Metzger, T., and Decker, B.	Characteristics of Outreach to the Highest Risk: Violence Prevention in Chicago.	Conference paper

#### 5.1.2 Included studies

The search and screening resulted in a final selection of 16 studies, which met the inclusion criteria for this review. The 16 studies analysed 17 different interventions. Only five studies could be used in the data synthesis. Eight studies could not be used in the data synthesis as all reported outcomes were judged to have a critical risk of bias.

Three studies (Herrera et al., 2013; Kidd et al., 2020; and Arbreton & McClanahan, 2002) did not report data in a form that enabled the calculation of effect sizes and standard errors. We contacted the authors and data was kindly provided by author Scott Leon for the Kidd et al., 2020 study. Concerning the Herrera et al. (2013) study we made contact with author David Dubois who kindly is willing to provide the data, however we are still awaiting the data and the study will be included in the data synthesis in any future update of the review. Finally, concerning the study by Arbreton and McClanahan (2002) (analysing two different intervention with two different populations), we contacted the authors. The study was published by Public/Private Ventures (P/PV) and as P/PV ceased operations in 2012 the data is no longer available (mail correspondence with authors Amy Arbreton and Wendy McClanahan, 12 August 2021).

Further, one study (McClanahan et al., 2012), published by P/PV, did not provide enough information to assess risk of bias as it referred to a technical appendix for details concerning the statistical model. This technical appendix is unfortunately no longer available (according to correspondence with author Wendy McClanahan).

In Table 2 we show the total number of studies, that met the inclusion criteria for this review. The first column shows the total number of studies grouped by country of origin. The second column shows the number of these studies that did not provide enough data to calculate an effect estimate. The third column gives the number of studies that were coded with Critical risk of bias. The last column gives the total number of studies used in the data synthesis.

**Table 2:** Number of included studies by country

		Reduction due to		
Country	Total	Missing data	Critical risk of bias	Used in data synthesis
Canada	2	0	1	1
USA	14	3	7	4
Total	16	3	8	5

Eight studies were judged overall Critical risk of bias (see supplementary documents for the detailed risk of bias assessments). In accordance with the protocol, we excluded studies rated overall Critical risk of bias items from the data synthesis on the basis that they would be more likely to mislead than inform. Three studies did not provide enough information enabling us to calculate an effect size and standard error or did not provide enough information to assess risk of bias. All studies are listed in Table 3 along with the reason why the study was not used in the data synthesis.

**Table 3:** Characteristics of included studies

Study	Country	State/province	Outcome	Used in data synthesis/reason not used
Arbreton and McClanahan (2002)	USA	Data was collected at multiple sites in different states	Relationship behaviours, Positive use of leisure time, School behaviours, Delinquent behaviours and Gang behaviours.	Cannot calculate effect size and SE
Baer et al. (2007)	USA	Washington	Alcohol, abstinence and drug (marijuana and other drugs) use in last 30 days.	Used in data synthesis
Campie (2014)	USA	Massachusetts	Incarceration	Rated Critical risk of bias
Gautier (2020)	Canada	Québec	Functioning, illness severity, SUD severity, emergency room visits, hospitalisations and Housing stability	Rated Critical risk of bias
Gold and Mattick Hans (1974)	USA	Illinois	Attitudes towards delinquency and education, school drop out, employment	Rated Critical risk of bias
Herrera et al. (2013)	USA	Washington	Depression, parent trust, Social acceptance, Self‐perceptions of academic abilities, grades, Misconduct and Prosocial behaviour, Self‐worth, Hope, Emotional symptoms, Prosocial behaviour, Honesty, Conduct problems, School liking, Educational expectations	Cannot calculate effect size and SE
Kidd (2020)	Canada	Ontario	NEET status, housing status, mental health, addictions, quality of life, mindfulness, social support, community Integration, resilence and hope	Used in data synthesis
McClanahan et al. (2012)	USA	Pennsylvania	Arrests and convictions for violent crime	Could not assess risk of bias as too little information was provided
Peterson et al. (2006)	USA	Washington	Drug (other than marijuana) use in last 30 days.	Used in data synthesis
Spergel (2001)	USA	Data was collected at multiple states	Arrests (total arrests, violent arrests, property arrests, drug arrests, and other arrests (usually for minor offenses) and Self‐reports of the types and amount of offense (including gang‐related).	Rated Critical risk of bias
Spergel et al. (2003)	USA	Data was collected at multiple states	Outcomes from police records (total arrests, serious violence arrests, total violence arrests (serious and less serious), property arrests, drug arrests, and other arrests (usually for minor offenses)) and Self‐reported outcomes (offenses and gang involvement changes)	Rated Critical risk of bias
Spergel (2004a)	USA	Data was collected at multiple states	Arrests (police records) and Self‐reported gang‐membership and arrests	Rated Critical risk of bias
Spergel (2004b)	USA	Data was collected at multiple states	Arrests (police records) and Self‐reported gang‐membership and arrests	Rated Critical risk of bias
Thompson and Jason (1988)	USA	Illinois	Gang membership	Used in data synthesis
Walker et al. (2019)	USA	Washington	Warrants ordered	Used in data synthesis
Williams et al. (2002)	USA	Data was collected in USA, Canada and UK: cities of Toronto (Ontario), Boston (Massachusetts), and London, UK	Delinquency, School grades and self‐esteem	Rated Critical risk of bias

The main characteristics of the five studies used in the data synthesis are shown in Table 4.

**Table 4:** Characteristics of studies used in data synthesis

Characteristic		
Year start of intervention	Average (SD)	2005 (12.5)
	Range	1985–2017
Number of participants, treated	Average (SD)	116 (129.9)
	Range	30–346
Number of participants, control	Average (SD):	81 (76.5)
	Range	32–321
Number of participants, total	Average (SD)	269 (254.93)
	Range	62–629
Percent female	Average (SD)	41 (4.2)
	Range	35–45
Mean age	Average (SD)	17 (3.2)
	Range	13–22
Percent white (not reported in one study)	Average (SD)	43 (26.3)
	Range	20–72

**Number of studies (%)**		
Country	Canada	1 (20)
	USA	4 (80)
Target population	Homeless youth	3 (60)
	Youth at risk of failing to appear for court hearings	1 (20)
	Youth at risk of gang membership	1 (20)
Services	Mental health	1 (20)
	Peer support	1 (20)
	Case management	2 (40)
	Brief motivational intervention	2 (40)
	Afterschool activities (athletics programmes, job skills training workshops, social/recreational activities (e.g., pizza parties) and educational assistance)	1 (20)

The timespan in which included studies were carried out is 32 years, from 1985 to 2017 and on average, the intervention year was 2005. One study was carried out in Canada, the remaining in the USA. The average number of participants in outreach analysed was 116, ranging from 30 to 346 and the average number of controls was 81, ranging from 32 to 321. The average of outreach participants was 17 years ranging from 13 to 22 years. On average females constituted a little less than half of outreach participants, 41%. Ethnicity of outreach participants was reported in only four studies and the average percent of white was 43% with great variation, ranging from 20% to 72%. The target population was homeless youth in three studies and youth at risk of failing to appear for court hearings and youth at risk of gang membership in one study each. The services provided in connection with outreach were mental health (one study), peer support (one study), case management (two studies) brief motivational intervention (two studies) and after‐school activities (one study). Note that more than one activity could be provided in connection with outreach.

#### 5.1.3 Excluded studies

In addition to the 16 studies that met the inclusion criteria for this review, 17 studies at first sight appeared relevant but did not meet our criteria for inclusion. The studies and reasons for exclusion are given in Table 5. More than a third (seven studies) were excluded because the intervention analysed was not outreach as defined in this review. Other reasons were lack of control group (three studies), intervention not targeted to youth (two studies) and no analysis on an individual level were performed (five studies).

**Table 5:** Studies excluded with reason

Study	Reason for exclusion
Augimeri et al. (2007)	The SNAPTM under 12 outreach project (ORP) is a manualized 12‐week outpatient program with five primary components, not outreach
Campie et al. (2017)	City level outcomes only
Domina (2009)	It is a college preparation program for the disadvantaged, and the study provides little information on the sort of outreach activities available to the students and schools that participate in outreach, some of them may be talent programs and some offer offer yearround college advising and information, academic counselling, tutoring services, and special full‐day summer programs.
Georgiades (2003)	Not outreach as defined in this review
Green et al. (2011)	Not outreach as defined in this review
Guo and Slesnick (2017)	Both groups receive outreach followed by drop‐in or shelter
Hureau (2016)	Not targeted to youth but to gangs in general, age is not mentioned or reported at all
Lee (Date unknown)	School children are referred to a programme taken place both at school and at home
Lipman et al. (2008)	The SNAPTM under 12 outreach project (ORP) is a manualized 12‐week outpatient program with five primary components, not outreach
Office of Research, Evaluation, and Assessment's High School Evaluation Unit (1993)	Only Babygram is relevant and there is no control group
Petrosino (2017)	City level outcomes only
Power et al. (2007)	Evaluates only LEO which is not outreach and describes OASIS which is outreach but not as defined in the current review
Spicer et al. (2015)	Not targeted to youth: Participants ranged in age from 19 to 82 years (mean: 41 years). Target population is homeless in general
Webster et al. (2012)	Outcomes are homicides and nonfatal shootings and they compare areas with areas
Weiler (1994)	Only Babygram is relevant and there is no control group
Wilson et al. (2010)	Areas are compared on number of homicides and violence incidents and outreach is not targeted children and young people
Wilson and Chermak (2011)	Area analysis only

### 5.2 Risk of bias in included studies

The risk of bias coding for each of the 16 studies and their outcomes is shown in a supplementary document.

Four studies reported on randomised trials, all individually randomised trials.

Table 6 shows a summary of the risk of bias associated with the randomised studies.

**Table 6:** Summary risk of bias randomised studies

	Low risk of bias	Some concerns	High risk of bias	Unclear
Overall judgement	0	4	0	0
Randomisation process	0	4	0	0
Deviations from intervention	0	4	0	0
Missing outcome data	1	3	0	0
Measurement of outcome	0	4	0	0
Selection of reported results	1	3	0	0

Three studies reported an appropriate method of randomisation and to some extent showed or discussed baseline imbalances on the pre‐specified confounders. We rated all three studies Some concerns on the Randomisation Process item as they all had some issues with the balance on the pre‐specified confounders. One study did not report the randomisation method but most likely it was concealed and there were no imbalances on the pre‐specified confounders. This study was also rated Some concerns on the Randomisation Process item. On the Deviations from intervention item, all four studies were rated some concerns, mainly due to lack of blinding. Concerning missing outcome data, one study had no issues, and we rated it Low risk of bias, three studies were rated Some concerns. All four studies were rated Some concerns on the Measurement of Outcome item, mainly due to lack of blinding. One study was rated Low risk of bias on the Selection of Reported Results item, the remaining were rated Some concerns as there was no a priori analysis plan and an insufficient reporting of outcomes. Overall, none of the studies were rated Low risk of bias, they were all rated some concerns overall.

Unfortunately the study Herrera et al. (2013) did not report data in a form that permitted calculation of an effect size and standard error (see the supplementary document *Numerical outcome* for an explanation).

The assessment of one study (Walker et al., 2019) was rated using the ROBINS‐I tool, as even though participants were randomised (although not using an appropriate method), youth assigned to the comparison group from a previous evaluation (approximately 1 year before the current study) was included. This compromised the randomisation to an extent that it was most appropriate to assess the risk of bias using the ROBINS‐I tool.

The remaining 11 studies used non‐randomised designs, 1 study (Arbreton & McClanahan, 2002) reported on 2 different interventions including different individuals so in total 13 interventions were rated using the ROBINS‐I tool. Table 7 shows a summary of the risk of bias associated with the non‐randomised studies. As stated in the protocol, we stopped the assessment of a non‐randomised study outcome when it was rated ‘Critical’ on any of the items, therefore not all studies are rated on all domains. One study (McClanahan et al., 2012) stated that a detailed description of the analysis was summarised in the Technical Appendix, which unfortunately is not available. Thus, we could not assess risk of bias for this study, as there was very little (close to nothing) description in the main text. We contacted the author, but unfortunately she does not have the technical appendix and the publishing institution (P/PV) closed in 2012. The study could therefore not be rated.

**Table 7:** Summary risk of bias non‐randomised studies

	Low risk of bias	Moderate risk of bias	Serious risk of bias	Critical risk of bias	No information	Not rated
Overall judgement	0	0	4	8		1
Confounding bias	0	1	7	4	0	1
Selection bias	0	3	5	0	0	5
Classification bias	4	2	0	0	1	6
Deviation bias	4	1	1	0	1	6
Missing data	2	2	2	0	1	6
Measurement of Outcome	1	3	2	0	1	6
Selection of Reported Results	0	6	1	0	0	6

*Note*: Twelve studies were rated, one with two interventions, that is, 13 ratings in total, some rated differently on outcomes but best rating included here.

Eight of the non‐randomised studies were rated Critical risk of bias on the Overall judgement item corresponding to a risk of bias so high that the findings should not be considered in the data synthesis. The overall Critical risk of bias rating was mainly due to issues on the Confounding bias item; four were rated Critical risk of bias on this item; that is, they failed to establish a comparison group that was balanced on important confounders and further either did not control for any confounders or controlled for confounders but included only those participants who had an event (arrest) in either the pre‐programme period or in the programme period in the analysis of that particular outcome (arrests). The remaining four studies rated Critical risk of bias on the Overall judgement item, were rated Serious risk of bias on several other items (all four on the Confounding bias and Selection bias items and one study in addition on the Reporting item and two studies in addition on the Measurement item) which lead to an Overall judgement rating of Critical risk of bias.

Three studies (reporting on four interventions) were rated Serious risk of bias overall. Unfortunately, the study (Arbreton & McClanahan, 2002) reporting on two interventions did not report data that permitted calculation of an effect size and standard error. We contacted the authors but unfortunately, the data is no longer available as the publishing institution (P/PV) closed in 2012. This left only two non‐randomised studies to be used in the data synthesis.

Of the four interventions not rated Critical risk over bias overall, all had serious issues on this item. On the Selection bias item three were rated Moderate risk of bias and one was rated Serious risk of bias. All were rated Low risk of bias on the Classification item; three were rated Low risk of bias on the Deviation item and one was rated Moderate. On the missing data item two were rated Low risk of bias, one was rated Moderate and one was rated Serious risk of bias. On the measurement item, one was rated Low risk of bias and three were rated Moderate risk of bias. All four were rated Moderate risk of bias on the Selection of Reported Results mainly because there was no a priori analysis plan.

### 5.3 Effects of interventions

#### 5.3.1 Synthesis of results

Five studies were not rated Critical risk of bias and reported data that permitted calculation of an effects size and standard error and could thus be used in the meta‐analysis.

A large variety of different outcomes were reported in the studies (e.g., drug use, abstinence, housing, mental and physical health).

To carry out a meta‐analysis, every study must have a comparable effect size. We synthesise effects separately by type of outcome (conceptual outcomes as outlined in section ‘Types of outcomes measures’) and time point (end of intervention and follow up). Unfortunately each type of outcome was only reported in a small subset of studies (in many cases in only one single study). Thus, each meta analysis contains a very small number of effect sizes, at most two. The studies included in the meta‐analyses contribute only a single effect size to each analysis.

All continuous outcomes (effect sizes measured as Hedges g) were coded such that a larger effect size indicated better outcomes for the treated group. All binary outcomes (reported as odds ratio) were likewise coded such that a larger effect size indicated better outcomes for the treated group.

##### Primary outcomes

Two studies analysed the effect of outreach on three different substance uses in last 30 days: drug (other than marijuana), marijuana and alcohol. Both studies reported on outcomes at two time points; 1 month post‐baseline and 3 months post‐baseline.

###### Drug, other than marijuana

**Figure 2:**
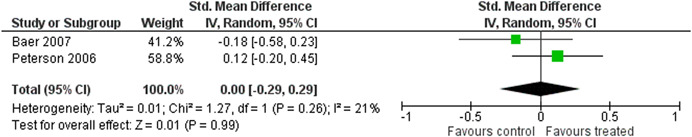
Analysis 1.1—1 month follow up

The random effects weighted standardised mean difference at 1 month post‐baseline was 0.0 (95% confidence interval [CI]: −0.29 to 0.29) and not statistically significant. The forest plot is displayed in Figure 2. There was a very small amount of heterogeneity between the studies; the estimated *τ* ^2^ was 0.01, *Q* = 1.27, *df* = 1 and *I* ^2^ was 21% as displayed in Figure 2.

**Figure 3:**
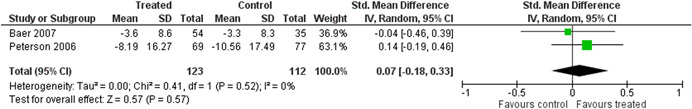
Analysis 1.2—3 months follow up

The random effects weighted standardised mean difference at 3 months post‐baseline was 0.07 (95% CI: −0.18 to 0.33) and not statistically significant. The forest plot is displayed in Figure 3. There was no heterogeneity between the studies; the estimated *τ* ^2^ was 0.00, *Q* = 0.41, *df* = 1 and *I* ^2^ was 0% as displayed in Figure 3.

###### Marijuana

**Figure 4:**
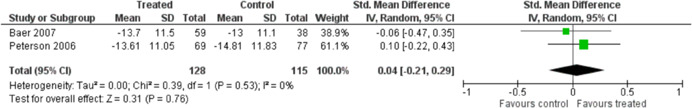
Analysis 2.1—1 month follow up

The random effects weighted standardised mean difference at 1 month post‐baseline was 0.04 (95% CI: −0.21 to 0.29) and not statistically significant. The forest plot is displayed in Figure 4. There was no heterogeneity between the studies; the estimated *τ* ^2^ was 0.00, *Q* = 0.39, *df* = 1 and *I* ^2^ was 0% as displayed in Figure 4.

**Figure 5:**
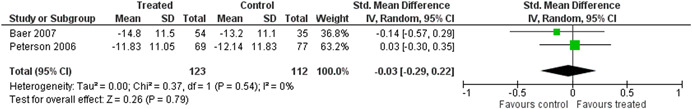
Analysis 2.2—3 months follow up

The random effects weighted standardised mean difference at 3 months post‐baseline was −0.03 (95% CI: −0.29 to 0.22) and not statistically significant. The forest plot is displayed in Figure 5. There was no heterogeneity between the studies; the estimated *τ* ^2^ was 0.00, *Q* = 0.37, *df* = 1 and *I* ^2^ was 0% as displayed in Figure 5.

###### Alcohol

**Figure 6:**
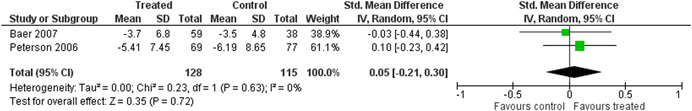
Analysis 3.1—1 month follow up

The random effects weighted standardised mean difference at 1 month post‐baseline was 0.05 (95% CI: −0.21 to 0.30) and not statistically significant. The forest plot is displayed in Figure 6. There was no heterogeneity between the studies; the estimated *τ* ^2^ was 0.00, *Q* = 0.23, *df* = 1 and *I* ^2^ was 0% as displayed in Figure 6.

**Figure 7:**
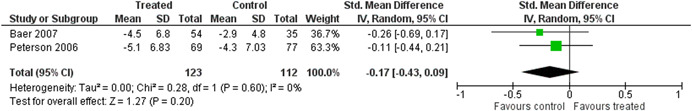
Analysis 3.2—3 months follow up

The random effects weighted standardised mean difference at 3 months post‐baseline was −0.17 (95% CI: −0.43 to 0.09) and not statistically significant. The forest plot is displayed in Figure 7. There was no heterogeneity between the studies; the estimated *τ* ^2^ was 0.00, *Q* = 0.28, *df* = 1 and *I* ^2^ was 0% as displayed in Figure 7.

###### Other primary outcomes

In addition, a number of outcomes were reported in a single study only. The outcomes were measures on housing situation, NEET status, gang membership, externalising problems and delinquency/criminal behaviour. The effect sizes and 95% CIs are reported in Table 8.

**Table 8:** Additional primary outcomes

Author (year)	Measure	Outcome	ES [95% CI]
			SMD [95% CI]
Kidd (2020)	Housing Security Scale	Housing security	0.02 [−0.47, 0.51]
Kidd (2020)	Housing Security Subscale	Subjective housing security (housing satisfaction and perception of housing stability)	0.19 [−0.30, 0.68]
Kidd (2020)	GAIN Short Screener	Externalising	0.05 [−0.44, 0.54]
Kidd (2020)	GAIN Short Screener	Substance Use (note not actual use)	0.10 [−0.39, 0.59]
Walker et al. (2019)	Court records	Warrant ordered in Case setting	−0.35 [−0.53, −0.17]
Walker et al. (2019)	Court records	Warrant ordered in Arraignment	0.19 [0.01, 0.37]
Baer et al. (2007)	Self‐reported, 1 month FU	Abstinence in last 30 days.	−0.34 [−0.77, 0.09]
Baer et al. (2007)	Self‐reported, 3 month FU	Abstinence in last 30 days.	−0.40 [−0.83, 0.03]
			OR [95% CI]
Kidd (2020)	Self‐reported	Housing	2.01 [0.31, 12.94]
Kidd (2020)	Self‐reported	NEET	2.30 [0.66, 8.06]
Thompson and Jason (1988)	Comparing names with gang rosters provided by gang members involved with BUILD's remediation program.	Gang member	7.49 [0.81, 69.32]

###### Secondary outcomes

A number of secondary outcomes were reported in a single study only. The outcomes were Quality of Life, internalising symptoms, mental health, resilience, hope, mindfulness, interpersonal and community relations and support. The effect sizes and 95% CIs are reported in Table 9.

**Table 9:** Additional secondary outcomes

Author (year)	Measure	Outcome	ES [95% CI]
			SMD [95% CI]
Kidd (2020)	Mental Health Continuum Short Form	Mental health	−0.15 [−0.64, 0.34]
Kidd (2020)	Cognitive and Affective Mindfulness Scale	Mindfulness	−0.34 [−0.85, 0.17]
Kidd (2020)	Community Integration Scale		0.13 [−0.36, 0.62]
Kidd (2020)	Community Integration Measure		−0.07 [−0.56, 0.42]
Kidd (2020)	Connor‐Davidson Resilience Scale	Resilience	−0.28 [−0.79, 0.23]
Kidd (2020)	Adult Hope Scale	Hope	−0.18 [−0.67, 0.31]
Kidd (2020)	GAIN Short Screener	Internalising	0.04 [−0.45, 0.53]
Kidd (2020)	Medical Outcomes Study	Emotional Supports	0.08 [−0.41, 0.57]
Kidd (2020)	Medical Outcomes Study	Tangible Supports	−0.08 [−0.57, 0.41]
Kidd (2020)	Medical Outcomes Study	Affectionate Supports	0.07 [−0.42, 0.56]
Kidd (2020)	WHO Quality of Life	Physical Health	0.06 [−0.43, 0.55]
Kidd (2020)	WHO Quality of Life	Psychological	0.05 [−0.44, 0.54]
Kidd (2020)	WHO Quality of Life	Social	−0.29 [−0.80, 0.22]
Kidd (2020)	WHO Quality of Life	Environment	−0.11 [−0.60, 0.38]

## 6 DISCUSSION

### 6.1 Summary of main results

Overall, there were too few studies included in any of the meta‐analyses in order for us to draw any conclusion concerning the effectiveness of outreach. At most, the results from two studies could be pooled in a single meta‐analysis. It was only possible to pool the outcomes drug (other than marijuana) use, marijuana use and alcohol use each at two different time points (1 and 3 months follow up). At 1 month follow up the weighted averages varied between zero and 0.05 and at three months follow up between −0.17 and 0.07. None of them were statistically significant.

### 6.2 Overall completeness and applicability of evidence

We included in total five studies in the data synthesis and of these, a maximum of two studies reported the same outcome and could be used in a specific meta‐analysis. This number is lower than the number of studies (16) meeting the inclusion criteria. The reduction was caused by three different factors.

Eight studies were judged to have a Critical risk of bias and, in accordance with the protocol, we excluded these from the data synthesis on the basis that they would be more likely to mislead than inform. One study provided very little (close to nothing) information on the method of analysis in the main text and referred to a technical appendix for this information. We contacted the author, but unfortunately she does not have the technical appendix and the publishing institution (P/PV) closed in 2012. The study could therefore not be rated and could not be used in the data synthesis. Finally, two studies (reporting on three different interventions) did not report effect estimates or provide data that would allow the calculation of an effect size.

If all the included studies had provided an effect estimate with lower risk of bias, the final list of useable studies in the data synthesis would have been larger, which again would have provided a more robust literature on which to base conclusions.

All studies used in the data synthesis were from the USA and Canada. This narrow geographical coverage is a clear limitation of the review.

Long term follow‐up analyses were not possible. This is also a clear limitation of the review.

It was not possible to examine the impact of the moderators nor sensitivity analyses for each outcome to check whether the obtained results were robust across study design and methodological quality.

### 6.3 Quality of the evidence

The majority of studies (12) used non‐randomised designs, and four were randomised trials. Overall the risk of bias in the included studies was high. Among the non‐randomised studies only three studies (reporting on four interventions) were not rated Critical risk of bias (in addition, one study provided too little information to be rated). The level ‘Critical’ means: the study (outcome) is too problematic in this domain to provide any useful evidence on the effects of intervention, and it is excluded from the data synthesis.

None of the randomised trials were overall rated low risk of bias, they were all assessed to have some concerns overall.

We examined the risk of bias using Cochrane's revised risk of bias tool, ROB 2 (Higgins et al., 2019) for the randomised studies and using the model ROBINS–I, developed by members of the Cochrane Bias Methods Group and the Cochrane Non‐Randomised Studies Methods Group (Sterne et al., 2016) for the non‐randomised studies.

The quality of the evidence in this review was enhanced by excluding studies assessed to be at critical risk of bias using the ROBINS–I tool from the data synthesis. We believe this process excluded those studies that are more likely to mislead than inform.

With two studies contributing effect sizes for one outcome (although reported at two different time points) it is of little use to discuss overall consistency in the direction and magnitude of effects and heterogeneity between studies.

### 6.4 Potential biases in the review process

We performed a comprehensive electronic database search, combined with grey literature searching, and hand searching of key journals. All citations were screened in teams by two independent screeners from the review team (TPC, MCTM., FSB., and FLWS), and one review author (TF) assessed all included studies against inclusion criteria.

We believe that all the publicly available studies on the effect of outreach on young people's problem/high‐risk behaviour and social and emotional outcomes up to the censor date were identified during the review process.

However, six references were not obtained in full text.

We were unable to comment on the possibility of publication bias as at most two studies was included in the same meta‐analysis. Thus, we cannot rule out that there are still some missing studies, which were not published or made public.

We believe that there are no other potential biases in the review process as two members of the review team (MCTM, FLWS) independently coded the included studies. Any disagreements were resolved by discussion. Further, decisions about inclusion of studies were made by the two teams of each two members of the review team (TPC, MCTM, FSB, FLWS) and one review author (TF). Assessment of study quality and numeric data extraction was made by one review author (TF) and each study was checked by another review author (NTD).

### 6.5 Agreements and disagreements with other studies or reviews

One systematic review on outreach programmes for youth; only including programmes for street‐involved youth, a term used by the authors instead of homeless youth, was found (Connolly & Joly, 2012). The eligible participants were young people aged 12–25, who did not have a permanent place of residence. Eligible studies were articles published in peer‐review journals between 1990 and 2010 and there were no restrictions on how the studies measured an impact (i.e., studies without comparison groups were included). The only impact result reported is on later participation rates in the offered service. It is reported that outreach programmes successfully engage 63% of the street‐involved youth they connect with. It is not possible to compare this conclusion to the conclusions of the current review.

Further, four systematic reviews on street‐connected and/or homeless youth were located.

Coren et al. (2016), focused on street‐connected children and young people (i.e., living on, or closely connected to, the street), from birth to 24 years, and studies of harm reduction or reintegration interventions that used a comparison group study design were eligible. The searches were performed up to April 2015. The primary outcomes were inclusion and reintegration and the secondary outcomes were measures of health, well‐being and educational and occupational achievement. Thirteen studies were included and most of them compared therapy‐based services versus usual shelter and drop‐in services, or versus other therapeutic/health interventions. The authors conclude that no consistent results on a range of relevant outcomes within domains of psychosocial health, substance misuse and sexually risky behaviours were found and there was considerable heterogeneity between studies. Only two of the included studies compared an outreach approach to no treatment (the two studies comparing brief, motivational interventions to no treatment also included in the current review, Baer et al., 2007; and Peterson et al., 2006).

In the systematic review by Altena et al. (2010), studies published up to 2008 were included if they empirically examined the effectiveness of an intervention for homeless youth. There were no restrictions on study design, that is, before‐after studies were included. No meta‐analysis was performed, only a narrative analysis describing each study and results. The overall conclusion was ‘no compelling evidence of the effectiveness of interventions for homeless youth can be presented on the basis of the results (and the effect sizes) of these studies’. (p. 643). The conclusion concerning outreach approaches is based solely on two studies comparing brief, motivational interventions to no treatment also included in the current review.

In Slesnick et al. (2009), runaway, shelter, street or drop‐in centre recruited youth between the ages of 12–24 were included. The review also included studies assessing youth outcomes after shelter or drop‐in utilisation (i.e., service evaluations) and qualitative studies in addition to intervention studies. No meta‐analysis was performed, only a narrative analysis describing each study and results. When the searches were performed is not reported. The conclusion concerning outreach approaches is also based solely on the two studies comparing brief, motivational interventions to no treatment also included in the current review.

Studies that examined the effectiveness of interventions to improve substance abuse problems among homeless youth between the ages of 12 and 24 were included in Xiang (2013). Searches were performed up to April 2012. Only studies that reported data on substance use outcomes were included. All study designs, with or without a control group, that is, before‐after studies were included. No meta‐analysis was performed, only a narrative analysis describing each study and results. The conclusion concerning outreach approaches is based solely on the two studies comparing brief, motivational interventions to no treatment included in the current review.

In the current review, we focused on programmes with a targeted outreach component for youth aged 8–25. Participants needed not be homeless (but were eligible if they were), and we only included studies with a control group. However, as very few studies of a sufficiently low risk of bias and who further reported data that permitted calculation of an effects size and standard error were located, the conclusions in the current review do not disagree with the conclusions concerning outreach in the four systematic reviews described above.

## 7 AUTHORS' CONCLUSIONS

### 7.1 Implications for practice

Public as well as private after‐school programmes and youth clubs that provide healthy alternatives for youth may serve as important resources for reducing school failure and youth crime. However, it is questionable whether the youth who would benefit most are those who are attracted to and attend such programmes (Arbreton & McClanahan, 2002). Outreach work represents an important preventive working approach with the aim of attracting and serving the youth who are very unlikely to participate on their own and who probably need the help most.

Outreach programmes targeting at‐risk youth are designed to reach the youth who need help to prevent high‐school dropout, crime, drug abuse, and other forms of delinquency. Unfortunately the evidence on outreach programmes to improve life circumstances for at‐risk youth in OECD countries was inconclusive because too few studies could be used in the data synthesis (see section Overall completeness and applicability of evidence). Further, the few studies included in the data synthesis did not report results on the same type of outcome, leaving very few observations to base a conclusion. Only two studies reported on the same outcomes and could be pooled in meta‐analyses of drug and alcohol use. Furthermore, the two studies used in these meta‐analyses both had a very brief intervention called ‘Brief Motivational Intervention’ consisting of motivational interviewing (Baer et al., 2007; Peterson et al., 2006), in which participants were offered feedback on alcohol, marijuana, and other drug use frequency and perceived norms. Participants were provided with a ‘colourful booklet’ from which they could choose topics for discussion and the average session was 30 min (ranging from 10 to 70 min). In one study (Peterson et al., 2006) additional feedback sessions were offered to youths, but few were scheduled and none were completed. Given the multitude and severity of problems experienced by the participants in the two studies (e.g., homelessness, alcohol and illicit drug use) it does not seem surprising that the Brief Motivation Intervention offered was not comprehensive enough to engage the youth or make a real difference in the lives of the participants. Thus, the nature of the interventions used in the meta‐analysis also constitutes a severe limitation to the generalisability of findings, as it is possible that given the multitude and magnitude of problems experienced by at‐risk youth, there is a need for comprehensive multi‐component programmes in order for interventions to be effective.

Finally, all the available evidence used in the data synthesis was from either Canada or the USA, countries with a less developed welfare state and social security system (i.e., liberal regime countries) than for example the Scandinavian countries with comprehensive welfare state institutions (Esping‐Andersen, 1990). Thus, the findings may not be generalisable to other settings and systems outside North America.

### 7.2 Implications for research

Further research is required to fully address the effects of outreach on young people's problem/high‐risk behaviour. Few studies have investigated this issue using appropriate comparison groups, and none have investigated the long term effects. It should be acknowledged that research in this field is problematic for a number of practical and methodological reasons. Locating an appropriate control group is difficult as outreach programmes targeting at‐risk youth is a preventive working approach with the aim of attracting and serving the youth who are very unlikely to participate on their own and who probably need the help most (Arbreton & McClanahan, 2002). Further, as the aims of the programmes are typically to change the long‐term developmental paths of the participants, longitudinal studies are preferable.

We found only few randomised controlled trials and the risk of bias in most of the included non‐randomised studies was very high leaving only two non‐randomised studies to be used in the data synthesis.

These considerations point to the need for more rigorously conducted studies. Obtaining balance on important confounding factors may be difficult when participants are not randomised, which adds to the importance of statistically controlling for relevant factors.

Some studies reported only descriptive results even though data had been gathered on important confounding factors, such as age, ethnicity, risk factors and household characteristics The risk of bias due to confounding would be judged to be of less concern had the primary study authors controlled for these factors. As the data already are gathered it is recommended that analyses controlling for important confounding factors are carried out using these data. Unfortunately two of the included studies (one randomised and one non‐randomised) did not provide data that permitted the calculation of an effect size and standard error (and attempt to achieve them was fruitless) and could therefore not be used in the data synthesis.

Given the limited number of rigorous studies available from countries other than the USA and Canada, it would be natural to consider conducting randomised controlled trials even though it might be argued that it is difficult for the population of interest. However, depending on the specific target population, an appropriate control group could be obtained in several ways. If the target population is homeless youth, treatment and control youth may be recruited from drop‐in centres, shelters or other agencies serving homeless youth and to include an even broader range of youth street sampling methods could be used. All these techniques were for example combined in the randomised controlled study (Peterson et al. (2006)). If the target population is school children, schools could be the unit of randomisation or, as in the study Herrera et al. (2013) evaluating mentoring through the Big Brothers Big Sisters programme, In this study they reached at‐risk youth in a number of ways, including collaborations with schools, partnerships with social service agencies, participation in community activities and events, word of mouth, and other media/communications strategies. Interested youth were then randomised to treatment and a wait list control group.

Such adapted trials in other countries than the USA and Canada would have the potential of making useful contributions to the outreach effectiveness literature if due consideration is made to the strengths and weaknesses of the studies found in this review. Thus, besides specific attention would have to be paid to stringency in terms of conducting a well‐designed randomised trial with low risk of bias as well as ensuring that the sample sizes are large enough to enable sufficient power, the trials should also pay attention to reporting relevant outcomes with sufficient details for them to be included in an inverse variance weighted meta‐analysis of standardised effect sizes. Further, trials performed in countries with access to administrative data about the participant's school, housing, employment and health outcomes (e.g., Denmark) would enable the investigator to report on long‐term effects of the intervention.

## CONTRIBUTIONS OF AUTHORS

- Content: Trine Filges and Nina Thorup Dalgaard
- Systematic review methods: Trine Filges and Nina Thorup Dalgaard
- Statistical analysis: Trine Filges
- Information retrieval: Bjørn Christian Arleth Viinholt

## DECLARATIONS OF INTEREST

There are no potential conflicts of interest.

## PRELIMINARY TIMEFRAME

Approximate date for submission of the systematic review will be no longer than two years after protocol approval.

## PLANS FOR UPDATING THIS REVIEW

Trine Filges will be responsible for updating the review and updates can be expected each second year.

## DIFFERENCES BETWEEN PROTOCOL AND REVIEW

We planned to add a critical level of risk of bias to the RoB 2 tool with the same meaning as in the ROBINS‐I tool; that is, the study (outcome) is too problematic in this domain to provide any useful evidence on the effects of intervention, and it is excluded from the data synthesis. However, after publication of the protocol we became aware (through correspondence with Professor Julian Higgins) that our add‐on (of a ‘Critical’ risk of bias level) to the ROB 2 tool is in breach of the Creative Commons licence for RoB 2.

We therefore made the following change to the application of the ROB 2 tool:

In the case of a RCT, where there is evidence that the randomisation has gone wrong or is no longer valid, we planned to assess the risk of bias of the outcome measures using ROBINS‐I instead of ROB 2. Examples of reasons for assessing RCTs using the ROBINS‐I tool may include studies showing large and systematic differences between treatment conditions while not explaining the randomisation procedure adequately suggesting that there was a problem with the randomisation process; studies with large scale differential attrition between conditions in the sample used to estimate the effects; or studies selectively reporting results for some part of the sample or for only some measured outcomes. In such cases, differences between the treatment and control conditions are likely systematically related to other factors than the intervention and the random assignment is, on its own, unlikely to produce unbiased estimates of the intervention effects. Therefore, as ROBINS‐I allow for an assessment of for example confounding, we believe it is more appropriate to assess effect sizes from studies with a compromised randomisation using ROBINS‐I than ROB 2. We reported this decision as part of the risk of bias assessment of the outcome measure in question (one study and all outcomes measured in this study was moved from ROB 2 to ROBINS‐I). As other effect sizes assessed with ROBINS‐I, the effect sizes could have received a ‘Critical’ rating and thus be excluded from the data synthesis.

### 1 Search strategy deviations from protocol

We searched the bibliographical database SocIndex.

In January 2021, the Danish National Research Database: http://www.forskningsdatabasen.dk/, was discontinued as a joint search service for the local research databases of the Danish research institutions. Instead, we searched the following sources:

- Copenhagen Business School research portal: https://research.cbs.dk/
- Dansk Institut for Internationale studier ‐ DIIS' publikationer: https://www.diis.dk/publikationer
- Københavns Universitet forskning: http://forskning.ku.dk/find-en-forsker/
- Roskilde Universitets forskningsportal: https://forskning.ruc.dk/
- Syddansk Universitets forskningsportal: https://portal.findresearcher.sdu.dk/
- UC Viden ‐ Professionshøjskolernes Videndatabase: https://www.ucviden.dk/
- Aalborg Universitets forskningsportal: https://vbn.aau.dk/
- Aarhus Universitets forskningspublikationer: https://pure.au.dk/portal/

## DATA AND ANALYSES

**1 Drug (other than marijuana) use in last 30 days**

**Table:** 

**Outcome or Subgroup**	**Studies**	**Participants**	**Statistical Method**	**Effect Estimate**
1.1 1 month follow up	2	243	Std. Mean Difference (IV, Random, 95% CI)	0.00 [−0.29, 0.29]
1.2 3 months follow up	2	235	Std. Mean Difference (IV, Random, 95% CI)	0.07 [−0.18, 0.33]

**2 Drug (Marijuana) use last 30 days**

**Table:** 

**Outcome or Subgroup**	**Studies**	**Participants**	**Statistical Method**	**Effect Estimate**
2.1 1 month follow up	2	243	Std. Mean Difference (IV, Random, 95% CI)	0.04 [−0.21, 0.29]
2.2 3 months follow up	2	235	Std. Mean Difference (IV, Random, 95% CI)	−0.03 [−0.29, 0.22]

**3 Alcohol use last 30 days**

**Table:** 

**Outcome or Subgroup**	**Studies**	**Participants**	**Statistical Method**	**Effect Estimate**
3.1 1 month follow up	2	243	Std. Mean Difference (IV, Random, 95% CI)	0.05 [−0.21, 0.30]
3.2 3 months follow up	2	235	Std. Mean Difference (IV, Random, 95% CI)	−0.17 [−0.43, 0.09]

## SOURCES OF SUPPORT

Internal sources

- No sources of support provided
  External sources
- VIVE Campbell, Denmark

## Supporting information

Supporting information.

Supporting information.

Supporting information.

Supporting information.
