# Supplementary material for: Outreach programs to improve life circumstances and prevent further adverse developmental trajectories of at‐risk youth in OECD countries: A systematic review
Source: Campbell Syst Rev. 2022 Oct 17;18(4):e1282. doi: 10.1002/cl2.1282 (PMC9577261; doi:10.1002/cl2.1282)
Supplement: Supplementary file 1 — Supporting information. [file CL2-18-e1282-s004.docx]

# Appendices

## 1 Assessment of risk of bias in included studies

### User guide for unobservables

Systematic baseline differences between groups can compromise comparability between groups. Baseline differences can be observable (e.g. age and gender) and unobservable (to the researcher; e.g. motivation and ‘ability’). There is no single non-randomised study design that always solves the selection problem. Different designs solve the selection problem under different assumptions and require different types of data. Especially how different designs deal with selection on unobservables varies. The “right” method depends on the model generating participation, i.e. assumptions about the nature of the process by which participants are selected into a programme.

As there is no universal correct way to construct counterfactuals we will assess the extent to which the identifying assumptions (the assumption that makes it possible to identify the counterfactual) are explained and discussed (preferably the authors should make an effort to justify their choice of method). We will look for evidence that authors using e.g. (this is NOT an exhaustive list):

**Natural experiments:**

Discuss whether they face a truly random allocation of participants and that there is no change of behaviour in anticipation of e.g. policy rules.

**Matching (including propensity scores):**

Explain and discuss the assumption that there is no selection on unobservables, only selection on observables.

**(Multivariate, multiple) Regression:**

Explain and discuss the assumption that there is no selection on unobservables, only selection on observables. Further discuss the extent to which they compare comparable people.

**Regression Discontinuity (RD):**

Explain and discuss the assumption that there is a (strict!) RD treatment rule. It must not be changeable by the agent in an effort to obtain or avoid treatment. Continuity in the expected impact at the discontinuity is required.

**Difference-in-difference (Treatment-control-before-after):**

Explain and discuss the assumption that the trends in treatment and control groups would have been parallel, had the treatment not occurred.

### Justification of exclusion of studies using an instrumental variable (IV) approach

Studies using instrument variables (IV) for causal inference in non-randomised studies will not be included as the interpretation of IV estimates is challenging. IV only provides an estimate for a specific group namely, people whose behaviour change due to changes in the particular instrument used. It is not informative about effects on never-takers and always-takers because the instrument does not affect their treatment status. The estimated effect is thus applicable only to the subpopulation whose treatment status is affected by the instrument. As a consequence, the effects differ for different IVs and care has to be taken as to whether they provide useful information. The effect is interesting when the instrument it is based on is interesting in the sense that it corresponds to a policy instrument of interest. Further, if those that are affected by the instrument are not affected in the same way the IV estimate is an average of the impacts of changing treatment status in both directions, and cannot be interpreted as a treatment effect. To turn the IV estimate into a LATE requires a monotonicity assumption. The movements induced by the instrument go in one direction only, from no treatment to treatment. The IV estimate, interpreted as a LATE, is only applicable to the complier population, those that are affected by the instrument in the ‘right way’. It is not possible to characterise the complier population as an observation’s subpopulation cannot be determined and defiers do not exist by assumption.

In the binary-treatment–binary-instrument context, the IV estimate can, given monotonicity, be interpreted as a LATE; i.e. the average treatment effect for the subpopulation of compliers. If treatment or instruments are not binary, interpretation becomes more complicated. In the binary-treatment–multivalued-instrument (ordered to take values from 0 to *J*) context, the IV estimate, given monotonicity, is a weighted average of pairwise LATE parameters (comparing subgroup *j* with subgroup *j*−1). The IV estimate can thus be interpreted as the weighted average of average treatment effects in each of the *J* subgroups of compliers. In the multivalued-treatment (ordered to take values from 0 to *T*) – multivalued-instrument (ordered to take values from 0 to *J*) context, the IV estimate for *each pair of instrument values*, given monotonicity, is a weighted average of the effects from going from *t*-1 to *t* for persons induced by the change in the value of the instrument to move from any level below *t* to the level *t* or any level above. Persons can be counted multiple times in forming the weights.

*Bibliography*

Angrist, J.D., & Pischke, J.S. (2009*). Mostly Harmless Econometrics: An Empiricist’s Companion.* Princeton, NJ: Princeton University Press.

Heckman, J.J. & Urzúa, S. (2010). Comparing IV with structural models: What simple IV can and cannot identify. *Journal of Econometrics, 156*, 27-37.

Heckman, J.J., Urzúa, S. & Vytlacil, E. (2006). Understanding instrumental variables in models with essential heterogeneity. *The Review of Economics and Statistics, 88*(3), 389-432.

## 2 Search documentation

**Searches on bibliographical databases**

*Academic Search Premier. Search Performed 29/09/2020. Interface - EBSCOhost Research Databases. Search Screen - Advanced Search. Search modes - Boolean/Phrase.*

| **Search** | **Search Terms** | **Results** |
| --- | --- | --- |
| S9 | S4 AND S8 | 1,616 |
| S8 | S5 OR S6 OR S7 | 758,368 |
| S7 | AB ("at-risk" OR "school dropout*" OR "school failure*" OR "mental disorder*" OR abus* OR poverty OR crim* OR homeless* OR street* OR detached* OR delinquen*) AND AB (child* OR youth OR adolescent* OR young OR teen* OR student*) | 297,960 |
| S6 | TI (at-risk OR dropout* OR "school failure*" OR "mental disorder*" OR abus* OR poverty OR crim* OR homeless* OR street* OR detached* OR delinquen* OR detach*) | 530,091 |
| S5 | DE "AT-risk youth" OR DE "SCHOOL dropout prevention" | 1,941 |
| S4 | S1 OR S2 OR S3 | 20,426 |
| S3 | AB (outreach* OR "street work" OR "fieldwork" OR "youth work") AND AB (program* OR service* OR mentor* OR "social worker*" OR initiative* OR project*) | 15,834 |
| S2 | TI (outreach* OR "street work" OR "fieldwork" OR "youth work") | 6,169 |
| S1 | DE "OUTREACH programs" | 1,908 |

*ECONLIT. Search Performed 29/09/2020. Interface - EBSCOhost Research Databases. Search Screen - Advanced Search. Search modes - Boolean/Phrase.*

| **Search** | **Search Terms** | **Results** |
| --- | --- | --- |
| S7 | S5 AND S6 | 73 |
| S6 | S1 OR S2 | 841 |
| S5 | S3 OR S4 | 64,283 |
| S4 | AB ("at-risk" OR "school dropout*" OR "school failure*" OR "mental disorder*" OR abus* OR poverty OR crim* OR homeless* OR street* OR detached* OR delinquen*) AND AB (child* OR youth OR adolescent* OR young OR teen* OR student*) | 7,726 |
| S3 | TI (at-risk OR dropout* OR "school failure*" OR "mental disorder*" OR abus* OR poverty OR crim* OR homeless* OR street* OR detached* OR delinquen* OR detach*) | 58,834 |
| S2 | AB (outreach* OR "street work" OR "fieldwork" OR "youth work") AND AB (program* OR service* OR mentor* OR "social worker*" OR initiative* OR project*) | 658 |
| S1 | TI (outreach* OR "street work" OR "fieldwork" OR "youth work") | 229 |

*ERIC. Search Performed 29/09/2020. Interface - EBSCOhost Research Databases. Search Screen - Advanced Search. Search modes - Boolean/Phrase.*

| **Search** | **Search Terms** | **Results** |
| --- | --- | --- |
| S8 | S4 AND S7 | 906 |
| S7 | S5 OR S6 | 75,652 |
| S6 | AB ("at-risk" OR "school dropout*" OR "school failure*" OR "mental disorder*" OR abus* OR poverty OR crim* OR homeless* OR street* OR detached* OR delinquen*) AND AB (child* OR youth OR adolescent* OR young OR teen* OR student*) | 63,018 |
| S5 | TI (at-risk OR dropout* OR "school failure*" OR "mental disorder*" OR abus* OR poverty OR crim* OR homeless* OR street* OR detached* OR delinquen* OR detach*) | 30,195 |
| S4 | S1 OR S2 OR S3 | 10,526 |
| S3 | AB (outreach* OR "street work" OR "fieldwork" OR "youth work") AND AB (program* OR service* OR mentor* OR "social worker*" OR initiative* OR project*) | 6,531 |
| S2 | TI (outreach* OR "street work" OR "fieldwork" OR "youth work") | 1,722 |
| S1 | DE "OUTREACH programs" | 5,831 |

*SocIndex****.*** *Search Performed 29/09/2020. Interface - EBSCOhost Research Databases. Search Screen - Advanced Search. Search modes - Boolean/Phrase.*

| **Search** | **Search Terms** | **Results** |
| --- | --- | --- |
| S9 | S4 AND S8 | 1,269 |
| S8 | S5 OR S6 OR S7 | 223,785 |
| S7 | AB ("at-risk" OR "school dropout*" OR "school failure*" OR "mental disorder*" OR abus* OR poverty OR crim* OR homeless* OR street* OR detached* OR delinquen*) AND AB (child* OR youth OR adolescent* OR young OR teen* OR student*) | 106,641 |
| S6 | TI (at-risk OR dropout* OR "school failure*" OR "mental disorder*" OR abus* OR poverty OR crim* OR homeless* OR street* OR detached* OR delinquen* OR detach*) | 156,873 |
| S5 | DE "AT-risk youth" OR DE "SCHOOL dropout prevention" | 424 |
| S4 | S1 OR S2 OR S3 | 6,768 |
| S3 | AB (outreach* OR "street work" OR "fieldwork" OR "youth work") AND AB (program* OR service* OR mentor* OR "social worker*" OR initiative* OR project*) | 5,151 |
| S2 | TI (outreach* OR "street work" OR "fieldwork" OR "youth work") | 2,059 |
| S1 | DE "OUTREACH programs" | 372 |

*PsycINFO. Search Performed 29/09/2020. Interface - EBSCOhost Research Databases. Search Screen - Advanced Search. Search modes - Boolean/Phrase.*

| **Search** | **Search Terms** | **Results** |
| --- | --- | --- |
| S9 | S4 AND S8 | 1,551 |
| S8 | S5 OR S6 OR S7 | 339,338 |
| S7 | AB ("at-risk" OR "school dropout*" OR "school failure*" OR "mental disorder*" OR abus* OR poverty OR crim* OR homeless* OR street* OR detached* OR delinquen*) AND AB (child* OR youth OR adolescent* OR young OR teen* OR student*) | 212,742 |
| S6 | TI (at-risk OR dropout* OR "school failure*" OR "mental disorder*" OR abus* OR poverty OR crim* OR homeless* OR street* OR detached* OR delinquen* OR detach*) | 178,350 |
| S5 | DE "At Risk Populations" | 37,978 |
| S4 | S1 OR S2 OR S3 | 8,695 |
| S3 | AB (outreach* OR "street work" OR "fieldwork" OR "youth work") AND AB (program* OR service* OR mentor* OR "social worker*" OR initiative* OR project*) | 7,535 |
| S2 | TI (outreach* OR "street work" OR "fieldwork" OR "youth work") | 1,892 |
| S1 | DE "OUTREACH programs" | 1,164 |

*International Bibliography of the Social Sciences (IBSS). Search performed 30/09/2020. Search performed through the ProQuest advanced search interface.*

| Search | Search Terms | Results |
| --- | --- | --- |
| S7 | (ti((outreach* OR "street work" OR "fieldwork" OR "youth work")) OR (ab((outreach* OR "street work" OR "fieldwork" OR "youth work")) AND ab((program* OR service* OR mentor* OR "social worker*" OR initiative* OR project*)))) AND (ti((at-risk OR detached*)) OR (ab(("at-risk" OR "school dropout*" OR "school failure*" OR "mental disorder*" OR abus* OR poverty OR crim* OR homeless* OR street* OR detached* OR delinquen*)) AND ab((child* OR youth OR adolescent* OR young OR teen* OR student*)))) | 198 |
| S6 | ti((at-risk OR detached*)) OR (ab(("at-risk" OR "school dropout*" OR "school failure*" OR "mental disorder*" OR abus* OR poverty OR crim* OR homeless* OR street* OR detached* OR delinquen*)) AND ab((child* OR youth OR adolescent* OR young OR teen* OR student*))) | 28,496 |
| S5 | ti((outreach* OR "street work" OR "fieldwork" OR "youth work")) OR (ab((outreach* OR "street work" OR "fieldwork" OR "youth work")) AND ab((program* OR service* OR mentor* OR "social worker*" OR initiative* OR project*))) | 6,868 |
| S4 | ab(("at-risk" OR "school dropout*" OR "school failure*" OR "mental disorder*" OR abus* OR poverty OR crim* OR homeless* OR street* OR detached* OR delinquen*)) AND ab((child* OR youth OR adolescent* OR young OR teen* OR student*)) | 26,474 |
| S3 | ti((at-risk OR detached*)) | 2,451 |
| S2 | ab((outreach* OR "street work" OR "fieldwork" OR "youth work")) AND ab((program* OR service* OR mentor* OR "social worker*" OR initiative* OR project*)) | 4,656 |
| S1 | ti((outreach* OR "street work" OR "fieldwork" OR "youth work")) | 2,535 |

*Sociological Abstracts. Search performed 30/09/2020. Search performed through the ProQuest advanced search interface*

| **Search** | **Search Terms** | **Results** |
| --- | --- | --- |
| S7 | (ti((outreach* OR "street work" OR "fieldwork" OR "youth work")) OR (ab((outreach* OR "street work" OR "fieldwork" OR "youth work")) AND ab((program* OR service* OR mentor* OR ("social worker" OR "social workers") OR initiative* OR project*)))) AND (ti((at-risk OR detached*)) OR (ab(("at-risk" OR ("school dropout" OR "school dropouts") OR ("school failure") OR ("mental disorder" OR "mental disorders") OR abus* OR poverty OR crim* OR homeless* OR street* OR detached* OR delinquen*)) AND ab((child* OR youth OR adolescent* OR young OR teen* OR student*)))) | 615 |
| S6 | ti((at-risk OR detached*)) OR (ab(("at-risk" OR ("school dropout" OR "school dropouts") OR ("school failure") OR ("mental disorder" OR "mental disorders") OR abus* OR poverty OR crim* OR homeless* OR street* OR detached* OR delinquen*)) AND ab((child* OR youth OR adolescent* OR young OR teen* OR student*))) | 73,706 |
| S5 | ti((outreach* OR "street work" OR "fieldwork" OR "youth work")) OR (ab((outreach* OR "street work" OR "fieldwork" OR "youth work")) AND ab((program* OR service* OR mentor* OR ("social worker" OR "social workers") OR initiative* OR project*))) | 8,31 |
| S4 | ab(("at-risk" OR ("school dropout" OR "school dropouts") OR ("school failure") OR ("mental disorder" OR "mental disorders") OR abus* OR poverty OR crim* OR homeless* OR street* OR detached* OR delinquen*)) AND ab((child* OR youth OR adolescent* OR young OR teen* OR student*)) | 72,238 |
| S3 | ti((at-risk OR detached*)) | 2,757 |
| S2 | ab((outreach* OR "street work" OR "fieldwork" OR "youth work")) AND ab((program* OR service* OR mentor* OR ("social worker" OR "social workers") OR initiative* OR project*)) | 6,655 |
| S1 | ti((outreach* OR "street work" OR "fieldwork" OR "youth work")) | 2,365 |

*Social Science Citation Index & Science Citation Index. Search performed 30/09/2020. Search performed the Web of Science advanced search interface.*

| **Search** | **Results** | **Search Terms** |
| --- | --- | --- |
| # 7 | 1,035 | #6 AND #3  *Indexes=SCI-EXPANDED, SSCI Timespan=All years* |
| # 6 | 258,446 | #5 OR #4  *Indexes=SCI-EXPANDED, SSCI Timespan=All years* |
| # 5 | 113,548 | AB=("at risk" OR "school dropout*" OR "school failure*" OR "mental disorder*" OR abus* OR poverty OR crim* OR homeless* OR street* OR detached* OR delinquen*) AND AB=(child* OR youth OR adolescent* OR young OR teen* OR student*) |
| # 4 | 172,382 | TI=(at  risk OR dropout* OR "school failure*" OR "mental disorder*" OR abus* OR poverty OR crim* OR homeless* OR street* OR detached* OR delinquen* OR detach*)  *Indexes=SCI-EXPANDED, SSCI Timespan=All years* |
| # 3 | 16,835 | #2 OR #1  *Indexes=SCI-EXPANDED, SSCI Timespan=All years* |
| # 2 | 11,872 | AB=(outreach* OR "street work" OR "fieldwork" OR "youth work") AND AB =(program* OR service* OR mentor* OR "social worker*" OR initiative* OR project*)  *Indexes=SCI-EXPANDED, SSCI Timespan=All years* |
| # 1 | 6,463 | TI=(outreach* OR "street work" OR "fieldwork" OR "youth work")  *Indexes=SCI-EXPANDED, SSCI Timespan=All years* |

**Searches on other resources**

*ProQuest Dissertations and Theses. Search Performed 29/09/2020. Interface - EBSCOhost Research Databases. Search Screen - Advanced Search. Search modes - Boolean/Phrase.*

| Search | Search Terms | Results |
| --- | --- | --- |
| S7 | (ti((outreach* OR "street work" OR "fieldwork" OR "youth work")) OR (ab((outreach* OR "street work" OR "fieldwork" OR "youth work")) AND ab((program* OR service* OR mentor* OR ("social worker" OR "social workers") OR initiative* OR project*)))) AND (ti((at-risk OR detached*)) OR (ab(("at-risk" OR ("school dropout" OR "school dropouts") OR ("school failure") OR ("mental disorder" OR "mental disorders") OR abus* OR poverty OR crim* OR homeless* OR street* OR detached* OR delinquen*)) AND ab((child* OR youth OR adolescent* OR young OR teen* OR student*)))) | 427 |
| S6 | ti((at-risk OR detached*)) OR (ab(("at-risk" OR ("school dropout" OR "school dropouts") OR ("school failure") OR ("mental disorder" OR "mental disorders") OR abus* OR poverty OR crim* OR homeless* OR street* OR detached* OR delinquen*)) AND ab((child* OR youth OR adolescent* OR young OR teen* OR student*))) | 52,837 |
| S5 | ab(("at-risk" OR ("school dropout" OR "school dropouts") OR ("school failure") OR ("mental disorder" OR "mental disorders") OR abus* OR poverty OR crim* OR homeless* OR street* OR detached* OR delinquen*)) AND ab((child* OR youth OR adolescent* OR young OR teen* OR student*)) | 51,281 |
| S4 | ti((at-risk OR detached*)) | 4,582 |
| S3 | ti((outreach* OR "street work" OR "fieldwork" OR "youth work")) OR (ab((outreach* OR "street work" OR "fieldwork" OR "youth work")) AND ab((program* OR service* OR mentor* OR ("social worker" OR "social workers") OR initiative* OR project*))) | 8,908 |
| S2 | ab((outreach* OR "street work" OR "fieldwork" OR "youth work")) AND ab((program* OR service* OR mentor* OR ("social worker" OR "social workers") OR initiative* OR project*)) | 8,406 |
| S1 | ti((outreach* OR "street work" OR "fieldwork" OR "youth work")) | 993 |

*EBSCO Open Dissertations. Search performed 21/04/2021. Interface - EBSCOhost Research Databases. Search Screen - Advanced Search. Search modes - Boolean/Phrase.*

| **Search** | **Search Terms** | **Search** |
| --- | --- | --- |
| S7 | S3 AND S6 | 210 |
| S6 | S4 OR S5 | 29,491 |
| S5 | AB ("at-risk" OR "school dropout*" OR "school failure*" OR "mental disorder*" OR abus* OR poverty OR crim* OR homeless* OR street* OR detached* OR delinquen*) AND AB (child* OR youth OR adolescent* OR young OR teen* OR student*) | 15,398 |
| S4 | TI (at-risk OR dropout* OR "school failure*" OR "mental disorder*" OR abus* OR poverty OR crim* OR homeless* OR street* OR detached* OR delinquen* OR detach*) | 17,531 |
| S3 | S1 OR S2 | 2,698 |
| S2 | AB (outreach* OR "street work" OR "fieldwork" OR "youth work") AND AB (program* OR service* OR mentor* OR "social worker*" OR initiative* OR project*) | 2,526 |
| S1 | TI (outreach* OR "street work" OR "fieldwork" OR "youth work") | 294 |

*Search strings on Urban Institute https://www.urban.org/*

| Search | Results |
| --- | --- |
| outreach (In research area: Adolescents and Youth) | 146 |
| outreach (In research area: Children) | 357 |
| outreach (In research area: Community Engaged Methods) | 13 |
| outreach (In research area: Crime and Justice) | 205 |
| outreach (In research area: Gender and Sexuality) | 42 |
| outreach (In research area: Housing and Housing Finance) | 450 |
| outreach (In research area: Poverty, Vulnerability, and the Safety Net) | 414 |
| outreach (In research area: Race and Ethnicity) | 169 |

*Search strings on SwePub—Academic publications at Swedish universities*

| **Search terms** | **Results** |
| --- | --- |
| "Outreach" | 398 |
| outreach AND risk | 51 |
| Outreach AND "at-risk" | 7 |
| Outreach AND Youth | 17 |
| Outreach AND Student | 15 |
| "street work" OR "youth work" | 30 |
| "field work" AND youth | 21 |
| "field work" AND (youth OR child* OR adolescent OR student*) | 90 |
| detached and risk | 47 |

*Search strings on NORA - Norwegian Open Research Archives*

| **Search terms** | **Results** |
| --- | --- |
| "Outreach" | 3206 |
| ("outreach" OR "street work" OR "youth work") AND ("child*" OR "teen" OR "adolescent" OR "student" OR "delinquent youth") | 520 |
| ("outreach" OR "street work" OR "youth work") AND ("youth" OR "child*" OR "homeless" OR "risk") | 223 |
| ("outreach" OR "street work" OR "youth work") AND ("youth" OR "adolescent" OR "child*" OR "gang" OR "violence") | 146 |
| ("outreach" OR "social work*" OR "youth program" OR "youth intervention") AND ("youth" OR "adolescent" OR "teen") AND "risk" | 12 |
| ("outreach" OR "youth work" OR "street work") AND ("youth" OR "adolescent" OR "child*" OR "teen") AND ("risk" OR "at risk") | 17 |
| ("field work") AND ("risk" OR "at risk") | 6 |
| ("field work") AND ("youth" OR "adolescent" OR "child*" OR "teen") | 6 |
| ("field work") AND ("youth" OR "adolescent" OR "child*" OR "teen") AND ("risk" OR "at risk") | 1 |

*Search strings on CRISTIN (Current Research Information System In Norway)*

| **Search terms** | **Results** |
| --- | --- |
| "outreach" AND "youth" | 7 |
| "outreach" AND "risk" | 5 |
| "field work" AND "youth" AND "risk" | 117 |
| "field work" AND ("youth" OR "child*" OR "adolescent" OR "teen*") AND "risk" | 342 |
| ("outreach" OR "street work" OR "field work" OR "youth work") AND ("youth" OR "child*" OR "adolescent" OR "teen*") AND "risk" | 491 |

*Search strings on Skolporten—Swedish Dissertations*

| **Search terms** | **Results** |
| --- | --- |
| Outreach (in 'forskning') | 7 |
| Youth AND risk (in 'forskning') | 2 |
| field work (in 'forskning') | 41 |
| street work (in 'forskning') | 3 |
| at-risk (in 'forskning') | 10 |
| youth work (in 'forskning') | 13 |

*Search strings on DIVA–Digital Scientific Archives*

| **Search terms** | **Results** |
| --- | --- |
| Outreach | 304 |
| "outreach" AND "youth" | 16 |
| "street work" | 4 |
| "youth work" | 28 |
| "fieldwork" AND "youth" | 112 |
| "fieldwork" AND "youth" AND "risk" | 8 |
| "youth" AND "risk" AND "program" OR "youth" AND "risk" AND "service" | 74 |
| "youth" AND "at risk" | 165 |
| "adolescent" AND "at risk" | 140 |
| ("child*" AND "at risk" AND "program") OR ("child*" AND "at risk" AND "service") | 38 |
| ("field work" AND "risk") OR "field work" AND "at risk") | 14 |
| detached AND risk | 36 |
| "social work*" AND youth AND "at risk" | 62 |

*Search strings on CORE—research outputs from international repositories*

| **Search terms** | **Results** |
| --- | --- |
| Outreach (in English, Norwegian, Swedish and Danish – the first 14 pages with 10 hits per page is screened) | 140 |
| ("outreach" OR "field work" OR "street work" OR "youth work" OR "detached work") AND ("youth" OR "child*" OR "teen*" OR "adolescent") AND ("risk" OR "at-risk" OR "at risk") AND ("program" OR "intervention") AND "quantitative" AND ("comparison" OR "control") (in English, Norwegian, Danish and  'undetermined' - the first 10 pages with 10 hits per page is screened e) | 100 |
| title:((outreach AND at-risk) AND (youth OR child* OR teen* OR adolescent) ) abstract:((outreach AND at-risk) AND (youth OR child* OR teen* OR adolescent) ) | 476 |

*Search strings on AIR - American Institutes for Research*

| **Search terms** | **Results** |  |
| --- | --- | --- |
| outreach | 82 |  |
| youth AND risk | 85 |  |
| (field work OR youth work OR street work) AND youth AND risk | 75 |  |
| (program OR intervention) AND youth AND risk | 37 |  |
| (program OR intervention) AND (youth OR child* OR teen* OR adolescent) AND risk | 5 |  |
| homeless AND youth AND risk | 9 |  |
| homeless AND youth AND (intervention OR program) | 6 |  |

*Search strings on MDRC - Manpower Demonstration Corporation*

| **Search terms** | **Results** |
| --- | --- |
| outreach | 100 |
| (youth OR child* OR teen*) AND (risk OR at-risk) | 100 |
| (street work OR youth work OR field work) AND (youth OR child* OR teen*) | 72 |
| (street work OR youth work OR field work) AND (youth OR child* OR teen*) AND (risk OR at-risk) | 29 |

*Search strings on Aalborg University  research publications*

| **Search terms** | **Results** |
| --- | --- |
| outreach | 14 |
| detached youth work | 0 |
| street work AND youth | 3 |
| youth work AND at-risk | 3 |
| field work AND youth AND at-risk | 1 |
| (street work OR youth work OR field work) AND (youth OR child OR teen OR adolescent) AND at-risk | 11 |

*Search strings on Aarhus University research publications*

| **Search terms** | **Results** |
| --- | --- |
| outreach | 56 |
| detached youth work | 0 |
| street work AND youth | 7 |
| youth work AND at-risk | 45 |
| field work AND youth AND at-risk | 2 |
| ("street work" OR "youth work" OR "field work") AND (youth OR child* OR teen* OR adolescent) AND "at-risk" | 132 |

*Search strings on Copenhagen Business School research portal*

| **Search terms** | **Results** |
| --- | --- |
| outreach | 9 |
| detached youth work | 0 |
| street work AND youth | 0 |
| youth work AND at-risk | 6 |
| field work AND youth AND at-risk | 0 |
| (street work OR youth work OR field work) AND (youth OR child OR teen OR adolescent) AND at-risk | 36 |

*Search strings on Dansk Institut for Internationale studier - DIIS' publications*

| **Search terms** | **Results** |
| --- | --- |
| outreach | 14 |
| "detached youth work" | 0 |
| "street work" AND youth | 0 |
| youth work AND at-risk | 0 |
| field work AND youth AND at-risk | 0 |
| (street work OR youth work OR field work) AND (youth OR child OR teen OR adolescent) AND at-risk | 0 |

*Search strings on UC Viden - Professionshøjskolernes Videndatabase*

| **Search terms** | **Results** |
| --- | --- |
| outreach | 14 |
| detached youth work | 2 |
| street work AND youth | 17 |
| youth work AND at-risk | 37 |
| field work AND youth AND at-risk | 6 |
| (street work OR youth work OR field work) AND (youth OR child OR teen OR adolescent) AND at-risk | 212 |

*Search strings on Københavns Universitet forskning*

| **Search terms** | **Results** |
| --- | --- |
| outreach | 70 |
| detached youth work | 0 |
| street work AND youth | 6 |
| youth work AND at-risk | 35 |
| field work AND youth AND at-risk | 1 |
| (street work OR youth work OR field work) AND (youth OR child OR teen OR adolescent) AND at-risk AND quantitative | 40 |

*Search strings on Roskilde Universitets forskningsportal*

| **Search terms** | **Results** |
| --- | --- |
| outreach | 130 |
| detached youth work | 62 |
| street work AND youth | 168 |
| youth work AND at-risk | 394 |
| field work AND youth AND at-risk | 313 |
| (street work OR youth work OR field work) AND (youth OR child OR teen OR adolescent) AND at-risk AND quantitative | 329 |

*Search strings on Syddansk Universitets forskningsportal*

| **Search terms** | **Results** |
| --- | --- |
| outreach | 67 |
| detached youth work | 0 |
| street work AND youth | 5 |
| youth work AND at-risk | 73 |
| field work AND youth AND at-risk | 1 |
| (street work OR youth work OR field work) AND (youth OR child OR teen OR adolescent) AND at-risk AND quantitative | 76 |

*Search on Public/Private Ventures (P/PV) publications*

Although P/PV has ceased operations, its publications is archived with the Foundation Center's IssueLab: <https://ppv.issuelab>. All available publications (231) were screened

*Search strings on Google Scholar*

| **Search terms** | **Results** |
| --- | --- |
| allintitle: outreach at-risk youth | 17 |
| allintitle: outreach disadvantaged youth | 2 |
| allintitle: outreach at-risk young | 8 |
| allintitle: outreach disadvantaged young | 0 |
| allintitle: outreach at-risk adolescent | 3 |
| allintitle: outreach disadvantaged adolescent | 0 |
| allintitle: outreach detached youth | 2 |
| allintitle: outreach detached young | 1 |
| allintitle: outreach detached adolescent | 0 |

## 3 First and second level screening

First level screening is on the basis of titles and abstracts. Second level is on the basis of full text

Reference id. No. :

Reviewers initials:

Source:

Year of publication:

Country/countries of origin:

Author(s):

The study will be excluded if one or more of the answers to question 1-3 are ‘No’. If the answers to question 1 to 3 are ‘Yes’ or ‘Uncertain’, then the full text of the study will be retrieved for second level eligibility. All unanswered questions need to be posed again on the basis of the full text. If not enough information is available, or if the study is unclear, the author of the study will be contacted if possible.

**Screening questions:**

1. Does the study focus on outreach work?

Yes - include

No – if no then stop here and exclude

Uncertain - include

Question 1 guidance:

The intervention in this review is outreach work which may also be termed detached youth work, street work or fieldwork. Outreach efforts with services only focusing on nutritional and medical care (e.g. testing for HIV) will be excluded.

2. Are the participants young people between 8 and 25 years of age living in OECD countries , who either have experienced or is at-risk of experiencing an adverse outcome such as school failure, drug use, participation in delinquent behaviours, runaway and homelessness?

Yes - include

No – if no then stop here and exclude

Uncertain - include

Question 2 guidance:

At-risk may be based on such indicators as the young person’s level of association with negative peers (e.g. negative attitudes toward school and poor educational outlook, gang members etc.), hanging out on the streets or in gang neighbourhoods, poor academic history, coming from a highly distressed or crisis ridden, low income family in a racially/ethnically segregated neighbourhood, and prior involvement in illegal and delinquent activities.

3. Is the report/article a quantitative evaluation study with a comparison condition?

Yes - include

No – if no then stop here and exclude

Uncertain - include

Question 3 guidance:

We are only interested in primary quantitative studies with a comparison group, where the authors have analysed the data. We are not interested in theoretical papers on the topic or surveys/reviews of studies of the topic. (This question may be difficult to answer on the base of titles and abstracts alone.)

## 4 Data extraction

| **Names of author(s)** |
| --- |
| **Title** |
| **Language** |
| **Journal** |
| **Year** |
| **Country** |
| **Target group** |
| **Participant characteristic (age, gender, ethnicity, risk factors )** |
| **Services combined with the outreach component** (e.g. case management or participation in community programmes or a continuum of comprehensive services including education, employment, and intensive supervision) |
| **Duration (**number of weeks, months or years**)** |
| **Intensity (**number of hours per week/month**)** |
| **Type of data used in study (administrative, questionnaire, other (specify))** |
| **Time period covered by analysis (divide into intervention and follow up)** |
| **Sample size (divide into treated/comparison)** |

**Outcome measures**

Instructions: Please enter outcome measures in the order in which they are described in the report. Note that a single outcome measure can be completed by multiple sources and at multiple points in time (data from specific sources and time-points will be entered later).

| # | Outcome  & measure | Reliability & Validity | Format | Direction | Pg# & notes |
| --- | --- | --- | --- | --- | --- |
| 1 |  | Info from:  Other samples  This sample  Unclear  Info provided: | Dichotomy  Continuous | High score or event is  Positive  Negative  Can’t tell |  |

* Repeat as needed

**OUT COME DATA**

**DICHOTOMOUS OUTCOME DATA**

| OUTCOME | TIME POINT (s) (record exact time from participation, there may be more than one, record them all) | SOURCE | VALID Ns | CASES | NON-CASES | STATISTICS | Pg. # & NOTES |
| --- | --- | --- | --- | --- | --- | --- | --- |
|  |  | Questionnaire  Admin data  Other (specify)  Unclear | Participation | Participation | Participation | RR (risk ratio)  OR (odds ratio)  SE (standard error)  95% CI  DF  P- value (enter exact p value if available)  Chi2  Other |  |
|  |  |  |  |  |  |  |  |
|  |  |  | Comparison | Comparison | Comparison |  |  |
|  |  |  |  |  |  |  |  |

Repeat as needed

**CONTINUOUS OUTCOME DATA**

| OUTCOME | TIME POINT (s) (record exact time from participation, there may be more than one, record them all) | SOURCE  (specify) | VALID Ns | Means | SDs | STATISTICS | Pg. # & NOTES |
| --- | --- | --- | --- | --- | --- | --- | --- |
|  |  | Questionnaire  Admin data  Other (specify)  Unclear | Participation | Participation | Participation | P  t  F  Df  ES  Other |  |
|  |  |  |  |  |  |  |  |
|  |  |  | Comparison | Comparison | Comparison |  |  |
|  |  |  |  |  |  |  |  |

*Repeat as need
